# 1D Silver Organochalcogenide Semiconductors: Color Tunable Luminescence, Polarized Emission, and Long-Range Exciton Diffusion

**DOI:** 10.1021/jacs.5c12551

**Published:** 2025-10-14

**Authors:** Tomoaki Sakurada, Nithin Pathoor, Takuma Matsumoto, Rattapon Khamlue, Petcharaphorn Chatsiri, Jan Valenta, Tadashi Kawamoto, Shun Omagari, William A. Tisdale, Watcharaphol Paritmongkol, Yeongsu Cho, Martin Vacha

**Affiliations:** † Department of Materials Science and Engineering, 13290 Institute of Science Tokyo, Ookayama 2-12-1, Meguro-ku, Tokyo 152-8552, Japan; ‡ Department of Chemical Engineering, 2167 Massachusetts Institute of Technology, Cambridge, Massachusetts 02139, United States; § Yokohama Technical Center, AGC Inc., Yokohama, Kanagawa 230-0045, Japan; ∥ Department of Materials Science and Engineering, School of Molecular Science and Engineering, Vidyasirimedhi Institute of Science and Technology (VISTEC), Rayong 21210, Thailand; ⊥ Department of Chemical Physics and Optics, Faculty of Mathematics and Physics, Charles University, Ke Karlovu 3, 12116 Prague, Czech Republic; # Department of Chemistry, University of Houston, Houston, Texas 77004, United States

## Abstract

Metal organochalcogenides (MOCs) represent a promising class of organic–inorganic hybrid semiconductors with unique light-matter interactions. Their hybrid nature enables extensive structural and optoelectronic tunability via ligand engineering. In this study, we systematically modulated the electronic properties of ligands using Cl and Me functional groups, achieving precise control over the optoelectronic properties of Ag-based MOCs. Structural analysis revealed that these MOCs adopt a one-dimensional (1D) chain structure with organic ligands surrounding a Ag-chalcogen core. Density functional theory (DFT) calculations demonstrated that MOCs exhibit characteristics of 1D semiconductors with strongly dispersive conduction and valence bands aligned along the crystal rod directions. Experimentally, the MOCs displayed bright luminescence, with peaks centered between 560 and 690 nm. The substitution of Cl with Me groups in the benzene ligands induced a red shift in both absorption and photoluminescence, corroborated by experimental and theoretical analyses. Further optical measurements indicated that the emission from the MOCs is strongly polarized along the chain directions. Notably, Se-based MOCs exhibited enhanced exciton diffusivity along the chain axis with a diffusion length of 130 nm, which is among the highest reported for covalent systems. The observed trend in carrier diffusivity among individual compounds is attributed to differences in the effective masses of the carriers, as determined by DFT calculations. Our findings offer valuable insights into the systematic structural and property tuning of hybrid semiconductors and highlight the unique characteristics of the 1D MOC family.

## Graphical abstract

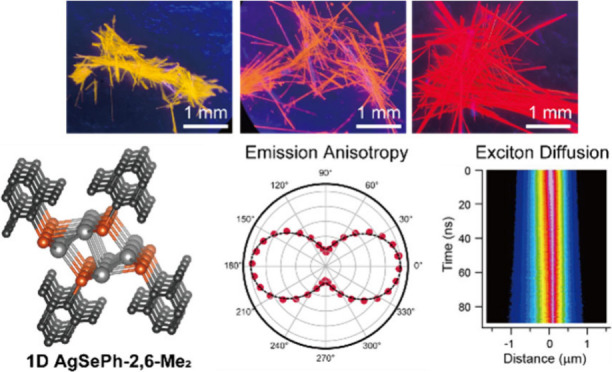

## Introduction

Metal organochalcogenides (MOCs) are an emerging class of hybrid semiconductors with applications spanning light emitters,^1–3^ sensors,^4–7^ and catalysts.^8,9^ MOCs are distinguished from other semiconductors, including halide perovskites and transition-metal dichalcogenides, by the covalent interaction between metals and organochalcogens. These interactions enable a diverse range of structural configurations, tunable optoelectronic properties, and excellent chemical stability.^10–13^

The hybrid nature of MOCs facilitates systematic tuning of their structural and optoelectronic properties through the selection of metals and organochalcogens.^14–19^ While coinage metals such as Cu­(I), Ag­(I), and Au­(I) are most comonnly used,^10–12^ other metals including Pb­(II), Cd­(II), and Ni­(II) have also been explored as potential candidates.^18^ Controlling the chalcogen type and their ratio has proven to be an effective strategy for tailoring optoelectronic properties, as demonstrated in studies on 2D silver phenylchalcogenides.^19–23^ Ligand modification is another promising way to fine-tune optoelectronic properties^14,23,24^ and to regulate morphology.^25–28^ Demessence and co-workers conducted a comprehensive study on morphology tuning of Au-based MOCs through ligand engineering.^29^ Their findings include enhanced PL quantum yield (PLQY),^2,30^ multiphase transitions triggered by external stimuli,^31^ and the formation of unique morphologies, such as a glass state.^32^ Similarly, Dou and co-workers demonstrated dimensional engineering of Pb based MOCs by changing the functional group of benzenethiols.^25^ These examples highlight the versatility and potential of MOCs as a platform for the design of hybrid semiconductors with tailored properties.

One-dimensional (1D) MOCs, composed of metal-chalcogen chains surrounded by organic ligands, have gained significant attention as luminescent materials (Table S1).^17,28,33–37^ For instance, Demessence and co-workers synthesized and evaluated the optical properties of Cu-, Ag-, and Au-based 1D MOCs^17^ and studied the effects of functional group positioning on benzenethiol ligands on the morphology of Ag-based MOCs.^17,36–38^ Hohman and co-workers examined the influence of ligands on Ag-based MOCs, finding that AgSPh-*o*-COOCH_3_ exhibited a PLQY of 22%, whereas AgSPh-*o*-OCH_3_ showed a PLQY of only 0–1%.^33^ These findings highlight the critical role of ligands in determining the structure and optoelectronic properties of 1D MOCs. Advances in structural engineering and further exploration of their emission properties are expected to uncover novel and intriguing features, driving deeper investigations into these promising materials.

Herein, we report a systematic study on seven 1D MOCs with *ortho*-functionalized benzenethiols or selenols, demonstrating wide tunability in PL emission peaks (560–690 nm) as well as unique optoelectronic properties such as strongly polarized emission and high exciton diffusivity. Structural analysis and density functional theory (DFT) calculations revealed that these MOCs exhibit characteristics of 1D semiconductors. For both S- and Se-based MOCs, substituting a methyl (Me) group for a chlorine (Cl) atom resulted in a narrower bandgap, as confirmed by both absorption edge measurements and DFT calculations. Orbital composition analysis indicated that the introduction of electron-withdrawing functional groups reduced the level of hybridization within the Ag-chalcogen cores, leading to an increase in the band gap of the 1D MOCs. Furthermore, we observed that Se-based MOCs exhibit higher exciton diffusivity compared to that of S-based MOCs. We attributed this to the lighter effective masses of carriers in the former, as determined by calculated band edges. These findings provide valuable guidelines for the systematic design and engineering of optoelectronic properties in covalently based hybrid semiconductors.

## Results and Discussion

### Synthesis and Characterization of MOCs

Recently, we reported that introducing fluorine atoms at the two *ortho*-positions of benzeneselenol induced a structural transformation from 2D blue-luminescent AgSePh to 1D yellow-emissive AgSePh-F_2_(2,6).^28^ Building on this insight, we synthesized seven Ag-based MOCs with systematically modified organic components by varying the functional groups and chalcogen elements (Figures 1a and 1d). Cl and Me groups were chosen as functional groups due to their electron-withdrawing and electron-donating properties, while maintaining comparable sizes (van der Waals radii: Cl, 1.75 Å; CH_3_, 1.91 Å).^39^ The organic ligands include two mono-*ortho*-substituted benzenethiols (**2-Cl** and **2-Me**), three di-*ortho*-substituted benzenethiols (**2,6-Cl**_**2**_, **2,6-Cl,Me**, and **2,6-Me**_**2**_) and two di-*ortho*-substituted benzeneselenols (**Se-2,6-Cl**_**2**_ and **Se-2,6-Me**_**2**_).

**Figure 1:**
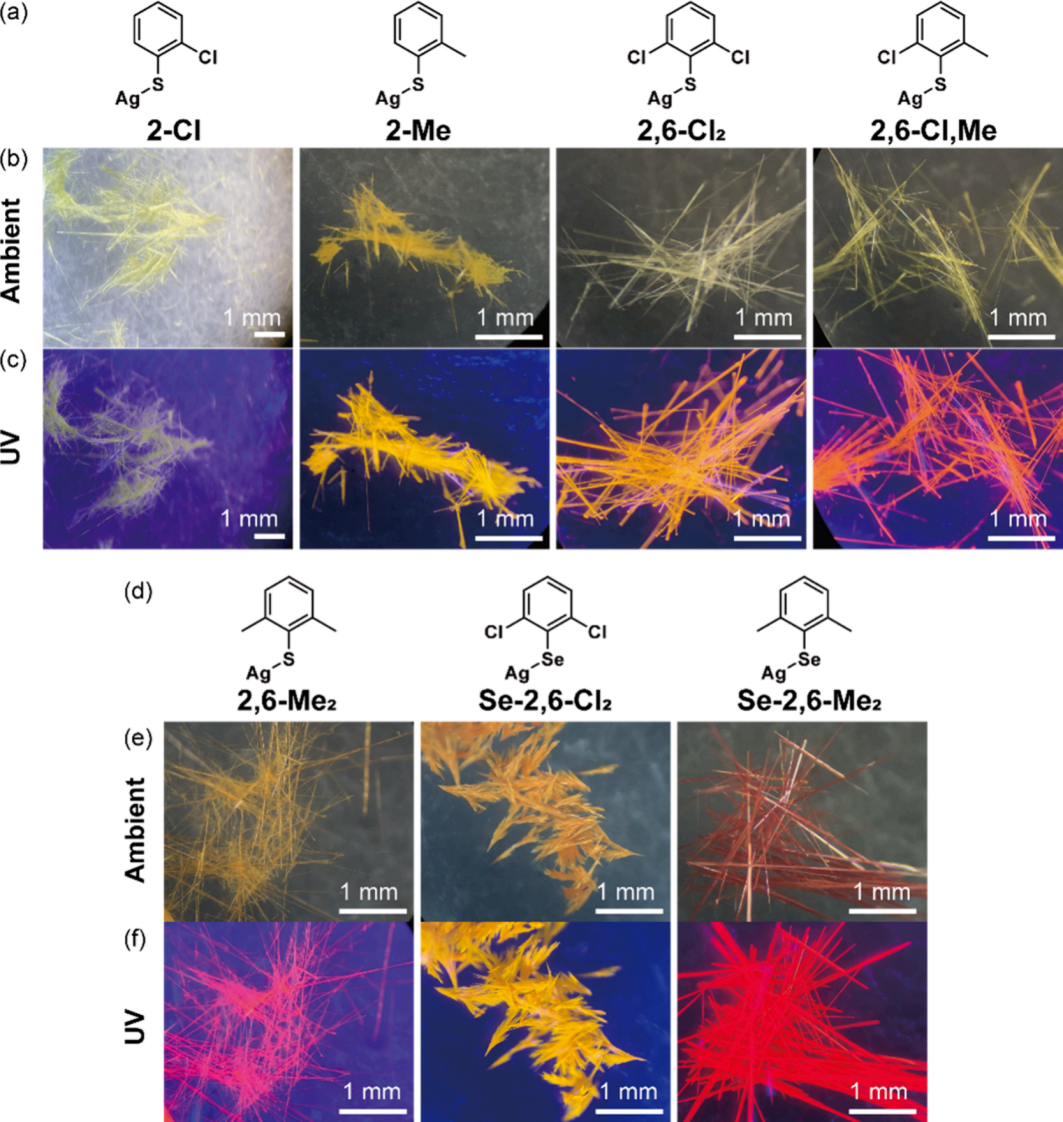
(a,d) Molecular structure of MOCs. Optical micrograph of MOCs (b,e) at ambient conditions and (c,f) under UV light.

First, we prepared organodiselenide precursors through the reaction of a Grignard reagent with elemental selenium.^40^ The resulting diselenides crystallized well, enabling single-crystal X-ray diffraction (SC-XRD) measurements (Table S2).

MOC crystals were prepared using an amine-assisted crystal growth method optimized for 1D MOCs.^28,41^ Briefly, a silver nitrate (AgNO_3_) solution was mixed with an organothiol or diselenide solution in 1-butylamine. The reaction mixture was placed in an uncapped vial, which was then placed inside a jar containing water. Slow diffusion of water and evaporation of solvents resulted in crystal formation in 2–3 days at room temperature (Figure S1). The crystals were subsequently washed with 2-propanol and toluene and dried under a vacuum. Millimeter-long needle-like crystals were obtained (Figures 1b and 1d). The morphology of each crystal was characterized by using SEM, revealing that the crystals consist of bundles of tiny crystals (Figure S2). Under UV excitation, all MOCs displayed luminescence ranging from yellow to red, depending on the ligand structure (Figures 1c and 1e). We observed that the color of the crystals changed uniformly, depending on the polarization angles, suggesting that the crystals comprise a single orientational domain (Figure S3).

SC-XRD analysis was conducted to elucidate the structures of the MOCs, excluding **2-Cl** (Table 1). For the analysis, suitable MOC crystals were grown of **2,6-Cl**_**2**_, **2,6-Cl,Me**, **2,6-Me**_**2**_, **Se-2,6-Cl**_**2**_, and **Se-2,6-Me**_**2**_ by chelation-driven recrystallization, which we recently established (see the Supporting Information for details).^42^ All MOCs exhibited a 1D chain structure composed of repeating Ag_4_S_4_ or Ag_4_Se_4_ units surrounded by organic ligands (Figure 2). Notable differences in core structures were observed between **2,6-Cl**_**2**_, **2,6-Cl,Me**, and **2,6-Me**_**2**_ (Figures 2a–f) compared to **2-Me**, **Se-2,6-Cl**_**2**_ and **Se-2,6-Me**_**2**_ (Figures 2g–l).

**Figure 2:**
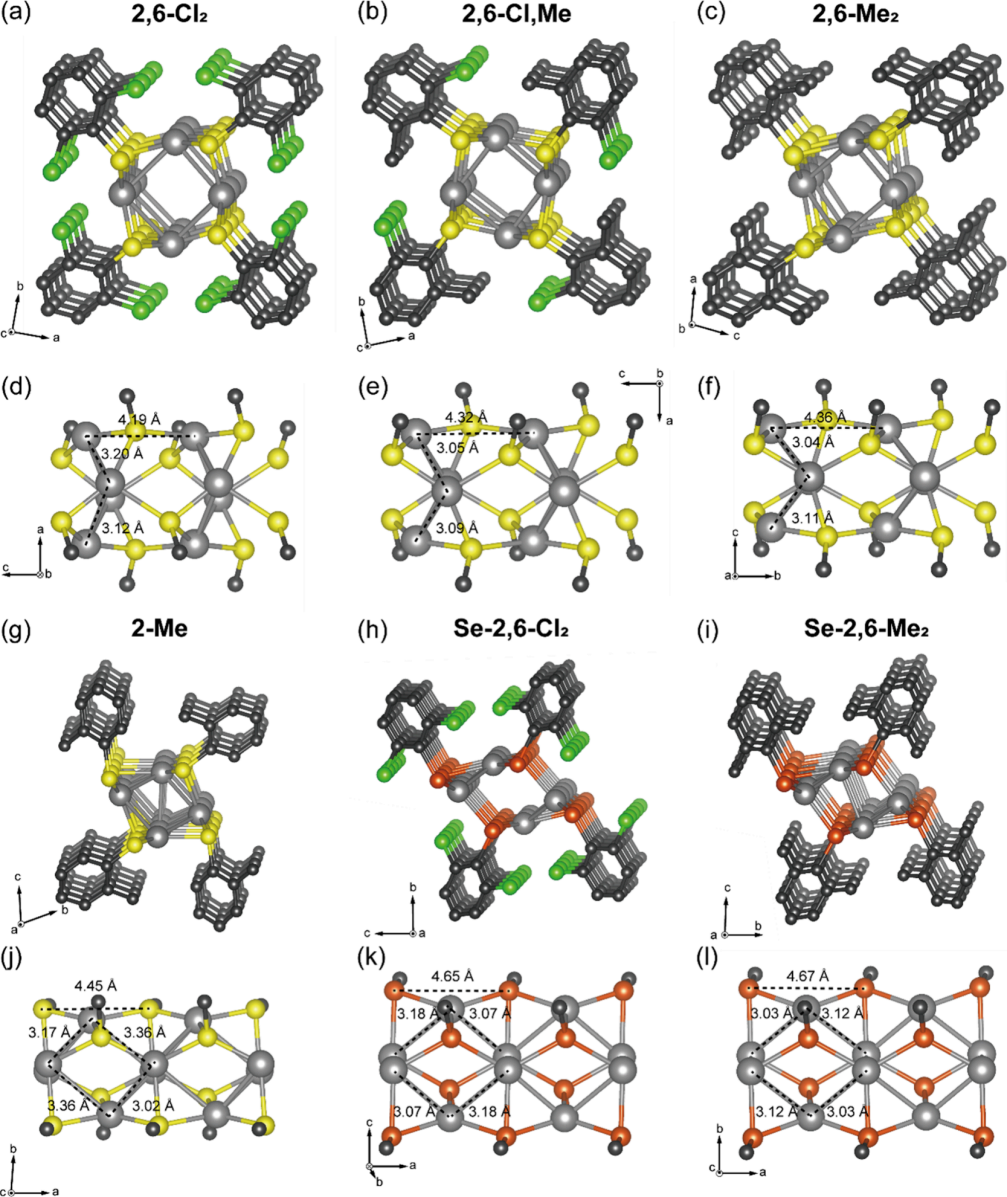
Crystal structures of (a) **2,6-Cl**_**2**_, (b) **2,6-Cl,Me**, (c) **2,6-Me**_**2**_, (g) **2-Me**, (h) **Se**-**2,6-Cl**_**2**_, and (i) **Se**-**2,6-Me**_**2**_. The Ag-chalcogen core structures of (d) **2,6-Cl**_**2**_, (e) **2,6-Cl,Me**, (f) **2,6-Me**_**2**_, (j) **2-Me**, (k) **Se**-**2,6-Cl**_**2**_, and (l) **Se**-**2,6-Me**_**2**_. Ag, S, Se, C, and Cl atoms are depicted by gray, yellow, orange, black, and light green spheres. Hydrogen and disordered atoms are omitted for clarity.

**Table 1:** Crystallographic Data for 1D MOCs

	**2-Me**	**2,6-Cl_2_**	**2,6-Cl,Me**	**2,6-Me_2_**	**Se-2,6-Cl_2_**	**Se-2,6-Me_2_**
Empirical formula	C_7_H_7_AgS	C_12_H_6_S_2_Cl_4_Ag_2_	C_14_H_12_S_2_Cl_2_Ag_2_	C_8_H_9_AgS	C_12_H_6_Ag_2_Cl_4_Se_2_	C_16_H_18_Ag_2_Se_2_
*M*_r_	231.06	571.83	531.00	245.08	665.63	583.96
Temperature (K)	113(2)	100	100	113(2)	100	100
Wavelength (Å)	0.71073	0.71073	0.71073	0.71073	0.71073	0.71073
Crystal system	Triclinic	Orthorhombic	Orthorhombic	Monoclinic	Monoclinic	Triclinic
Space group	*P*1̅	*P*2_1_2_1_2	*P*2_1_2_1_2	*P*2/*c*	*P*2_1_/*c*	*P*1̅
*a* (Å)	4.4595(2)	13.5008(11)	13.4148(10)	13.1195(9)	4.6495(9)	4.6660(3)
*b* (Å)	11.9202(5)	26.567(2)	26.608(2)	4.3577(4)	13.232(3)	12.2421(9)
*c* (Å)	13.9302(7)	4.1906(4)	4.3190(3)	27.2378(18)	24.381(5)	13.5623(9)
α (deg)	72.258(4)	90	90	90	90	87.676(2)
β (deg)	82.131(4)	90	90	99.459(7)	90.557(7)	88.551(2)
γ (deg)	84.123(4)	90	90	90	90	89.884(3)
*V* (Å^3^)	697.20(6)	1503.0(2)	1541.6(2)	1536.0(2)	1500.0(5)	773.82(9)
*Z*	4	4	4	8	4	2
Calculated density (Mg/m^3^)	2.201	2.527	2.288	2.120	2.948	2.506
Absorption coefficient (mm^–1^)	3.085	3.576	3.142	2.807	8.159	7.220
*F*(000)	448	1088	1024	960	1232	552
Crystal size (mm)	0.3 × 0.02 × 0.02	0.263 × 0.029 × 0.01	0.217 × 0.023 × 0.008	0.5 × 0.02 × 0.02	0.115 × 0.04 × 0.01	0.147 × 0.03 × 0.01
Theta range for data collection (deg)	2.694–30.650	2.751–27.455	2.156–27.538	1.998–30.670	2.272–18.494	2.198–27.530
Index ranges	–6 ≤ *h* ≤ 6, –17 ≤ *k* ≤ 16, –19 ≤ *l* ≤ 19	–17 ≤ *h* ≤ 17, –34 ≤ *k* ≤ 34, –4 ≤ *l* ≤ 5	–17 ≤ *h* ≤ 17, –34 ≤ *k* ≤ 31, –5 ≤ *l* ≤ 5	–16 ≤ *h* ≤ 18, –5 ≤ *k* ≤ 5, –37 ≤ *l* ≤ 38	–5 ≤ *h* ≤ 5, –16 ≤ *k* ≤ 16, –29 ≤ *l* ≤ 29	–5 ≤ *h* ≤ 6, –15 ≤ *k* ≤ 15, –17 ≤ *l* ≤ 17
Reflections collected	11277	16999	14605	15043	29269	25111
Independent reflections	4050, *R*_int_ = 0.0718	3452, *R*_int_ = 0.0440	3552, *R*_int_ = 0.0575	4471, *R*_int_ = 0.0512	2837, *R*_int_ = 0.0597	3499, *R*_int_ = 0.0516
Completeness to theta = 25.242° (%)	99.3	99.6	99.8	99.2	99.4	98.9
Data/restraints/parameters	4050/0/165	3452/0/182	3552/0/203	4471/0/185	2837/0/176	3499/0/186
Goodness-of-fit on *F* ^2^	1.021	1.153	1.150	1.125	1.149	1.166
Final *R* indices [*I* > 2σ(*I*)]	*R*1 = 0.0601, *wR*2 = 0.0729	*R*1 = 0.0388, *wR*2 = 0.0823	*R*1 = 0.0448, *wR*2 = 0.0990	*R*1 = 0.0413, *wR*2 = 0.0768	*R*1 = 0.0696, *wR*2 = 0.1318	*R*1 = 0.0312, *wR*2 = 0.0643
*R* indices (all data)	*R*1 = 0.1167, *wR*2 = 0.0827	*R*1 = 0.0516, *wR*2 = 0.0946	*R*1 = 0.0649, *wR*2 = 0.1182	*R*1 = 0.0691, *wR*2 = 0.1054	*R*1 = 0.0924, *wR*2 = 0.1512	*R*1 = 0.0458, *wR*2 = 0.0759
Largest diff. peak and hole (e Å^–3^)	–1.25 and 1.56	–1.22 and 0.95	–1.42 and 1.22	–1.75 and 1.00	–1.54 and 1.99	–1.41 and 1.45

In the cases of **2,6-Cl**_**2**_, **2,6-Cl,Me**, and **2,6-Me**_**2**_, the segmented Ag_4_S_4_ cores are connected by thiol ligands with slight variations in the lengths of Ag–Ag and Ag–S bonds. The distance between the segmented Ag_4_S_4_ cores ranges from 4.19 to 4.36 Å, increasing gradually with the substitution of Cl for Me groups (Figures 2d–f). In contrast, the cores of **2-Me**, **Se-2,6-Cl**_**2**_, and **Se-2,6-Me**_**2**_ are composed of Ag-chalcogen and Ag–Ag interactions throughout the chain length (Figures 2j–l). Despite the differences in functional groups and chalcogen species, these MOCs form an analogous structure.

The symmetry of the core differs between the two groups of MOCs. The chains of **2,6-Cl**_**2**_, **2,6-Cl,Me**, and **2,6-Me**_**2**_ are composed of two identical sheets rotated 180° around the chain direction, resulting in a symmetrical alignment along the crystalline orientation axis (Figure S4a). In contrast, the cores of **2-Me**, **Se-2,6-Cl**_**2**_, and **Se-2,6-Me**_**2**_ are formed by Ag-chalcogen sheets facing each other, which are both rotated and inverted, creating an inversion point at the center of the chain (Figure S4b).

Powder XRD patterns of ground crystals matched well with the simulated patterns from SC-XRD data, confirming the high purity of the synthesized MOCs (Figure S5). Thermogravimetric analysis showed that all MOCs are thermally stable up to 200 °C, with Cl-containing MOCs exhibiting higher stability compared to those with Me-substituted ligands (Figure S6). The mass fractions of the residues after heating to 400 °C indicate that all MOCs decomposed into metallic silver through thermolysis, consistent with previously reported Ag-based 1D MOCs (Figure S6 and Table S3).^17^

### Electronic Band Structure of MOCs

We performed DFT calculations to gain deeper insights into the electronic structure of MOCs (Figures 3 and S7) using the generalized gradient approximation (GGA). Among the MOCs studied, **2,6-Me**_**2**_, **Se-2,6-Me**_**2**_, and **Se-2,6-Cl**_**2**_ (Figures 3c,h,i) exhibit direct band gaps, whereas **2-Me**, **2,6-Cl,Me**, and **2,6-Cl**_**2**_ (Figures 3a,b,g) have indirect band gaps. The difference between the direct and indirect gaps is less than 0.04 eV (Table S4), indicating that the two gaps are nearly identical and have no significant effect on the subsequent analysis of band gap comparisons.

**Figure 3:**
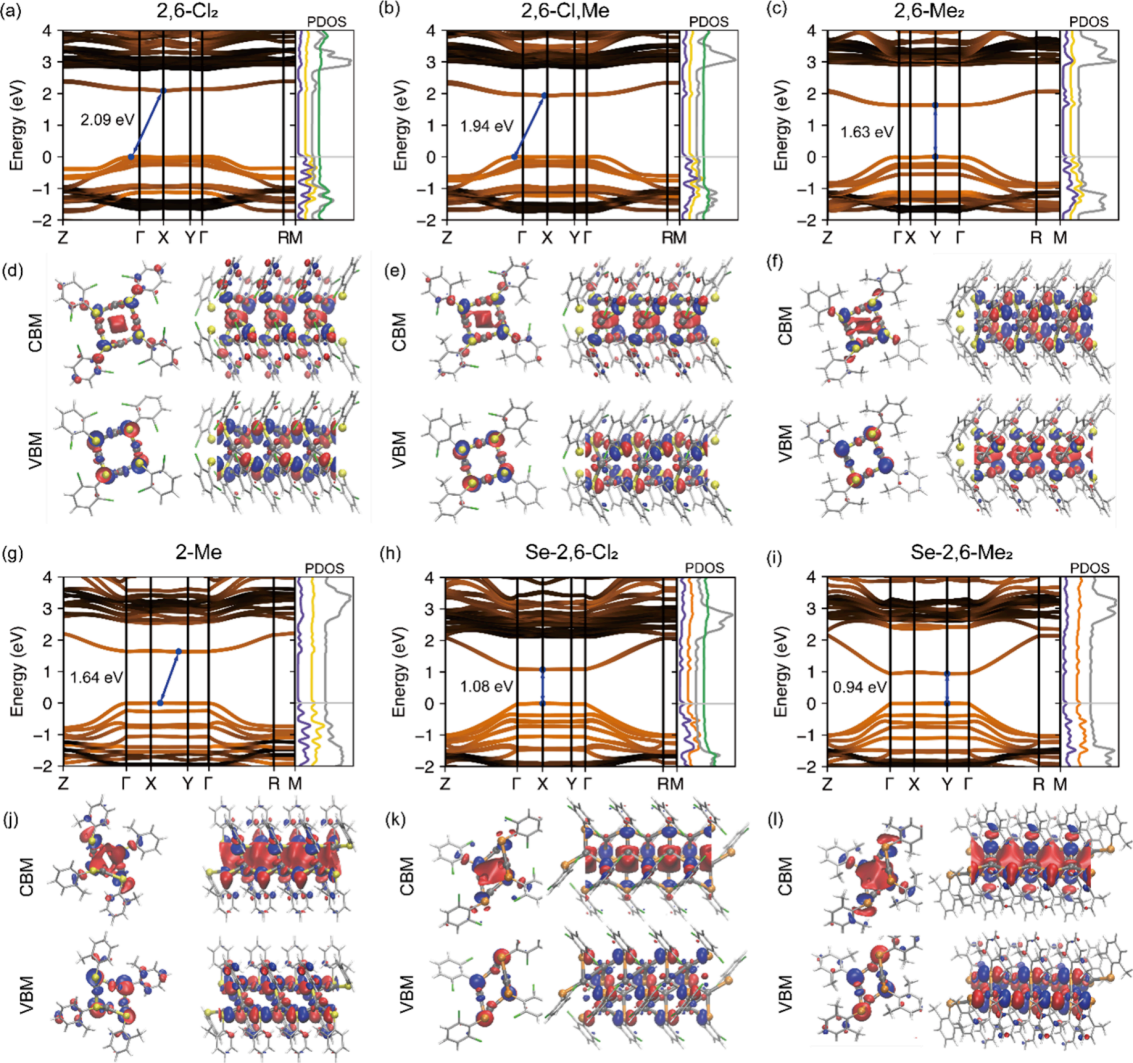
Calculated electronic band structure, projected density of states (PDOS), wave functions of conduction band minimum (CBM) and valence band maximum (VBM) of **2,6-Cl**_**2**_ (a,d), **2,6-Cl,Me** (b,e), **2,6-Me**_**2**_ (c,f), **2-Me** (g,j), **Se-2,6-Cl**_**2**_ (h,k), and **Se-2,6-Cl**_**2**_ (i,l). PDOS is shown for each atom: Ag (purple), S (yellow), Se (orange), C (gray), and Cl (light green). The blue and red surfaces depict the positive and negative phases of the wave function, respectively, with an isosurface value of 0.002 e/bohr^3^.

Across all MOCs, the valence band maximum (VBM) is composed of strongly hybridized Ag 4d and S 3p or Se 4p orbitals (Table S5). The conduction band minimum (CBM), on the other hand, features contributions from Ag 5s, Ag 4d, S 3s, S 3p or Se 3s, Se 3p, and C 2p orbitals, with the relative contributions from each orbital varying significantly among the different MOCs (Table S6).

When comparing S- and Se-containing MOCs such as **2,6-Cl**_**2**_ (Figure 3a) with **Se-2,6-Cl**_**2**_ (Figure 3h), and **2,6-Me**_**2**_ (Figure 3c) with **Se-2,6-Me**_**2**_ (Figure 3i), the Se-containing MOCs exhibit band gaps that are lower than those of their S-containing counterparts. Additionally, the ligand effect on the band gap of Se-containing MOCs is less pronounced than that on S-containing MOCs, with a gap difference of 0.14 eV between **Se-2,6-Cl**_**2**_ and **Se-2,6-Me**_**2**_, compared to 0.46 eV for **2,6-Cl**_**2**_ and **2,6-Me**_**2**_. This trend can be attributed to the positioning of the CBM in these MOCs. In Se-containing MOCs, the CBM is farther from the ligand-derived conduction bands, making the band gap less sensitive to ligand effects. This observation is consistent with findings for 2D AgSPh and AgSePh,^15^ where the selenide analog exhibits a smaller change in band gap.

The substitution of Cl with Me groups narrowed the band gap from 2.09 eV in **2,6-Cl**_**2**_ (Figure 3a) to 1.94 eV in **2,6-Cl,Me** (Figure 3b) to 1.63 eV in **2,6-Me**_**2**_ (Figure 3c), and from 1.08 eV in **Se-2,6-Cl**_**2**_ (Figure 3h) to 0.94 eV in **Se-2,6-Me**_**2**_ (Figure 3i). To understand the origin of the band gap trend, it is instructive to examine how the substituents influence orbital hybridization near the band edges. Among the S-containing MOCs with disubstituted ligands, the valence bands remain relatively similar, while the lowest conduction band becomes progressively less dispersive with an increasing number of Cl groups. The bandwidth decreases from 0.44 eV in **2,6-Me**_**2**_ to 0.40 eV in **2,6-Cl,Me** and 0.29 eV in **2,6-Cl**_**2**_ (Table S4). Concurrently, the contribution of the C 2p orbital to the CBM increases with the number of Cl groups, rising from 15% in **2,6-Me**_**2**_ (Figure 3c) to 26% in **2,6-Cl,Me** (Figure 3b), and 36% in **2,6-Cl**_**2**_ (Figure 3a). This increase in the contribution from the C 2p orbital is accompanied by a lower contribution from the S 3s and 3p orbitals (Table S6). The trend suggests that the more electron-withdrawing ligands draw electron density away from the S atoms, thereby reducing hybridization within the Ag_4_S_4_ core. The resulting decrease in hybridization leads to a narrower conduction band and an increased band gap. A similar increase in the band gap with higher electron-withdrawing ability was previously observed for 2D AgSPh.^14^

To gain insight into the carrier behavior in the crystals, we analyzed the band edges and calculated the effective masses of holes (*m*_h_*) and electrons (*m*_e_*) (Figure S8). Among the MOCs, **Se-2,6-Cl**_**2**_, and **Se-2,6-Me**_**2**_ exhibit lighter effective masses for both electrons and holes along the chain length, with *m*_h_* of ∼0.4*m*_0_ and *m*_e_* of 0.2*m*_0_, where *m*_0_ is the electron rest mass. The *m*_h_* of **2-Me** is comparable to that of Se-based MOCs, while the *m*_e_* is higher, at approximately 2*m*_0_. In contrast, **2,6-Cl**_**2**_, **2,6-Cl**,**Me**, and **2,6-Me**_**2**_ exhibit relatively flat band structures, resulting in larger effective masses with *m*_e_* of ∼5*m*_0_ and *m*_h_* of ∼1*m*_0_.

### Optical Properties of MOCs

We conducted absorption and PL emission studies to validate the theoretical calculations. The optical absorption of the 1D MOC crystals was measured by diffuse reflectance spectroscopy (Figure 4a). The MOC crystals exhibited broad absorption features, with peaks centered from 400 to 550 nm (Table 2). Under UV excitation, all MOCs displayed PL with peaks centered between 560 and 690 nm at room temperature (Figure 4a and Table 2). This luminescence covers a wide region from yellow to red in the Commission internationale de l’éclairage (CIE) color space, located at the edge of the chromaticity diagram (Figure S9). Substitution of Cl atoms with Me groups caused a red shift in both absorption and PL spectra for both S-based and Se-based MOCs, consistent with the theoretical calculations.

**Figure 4:**
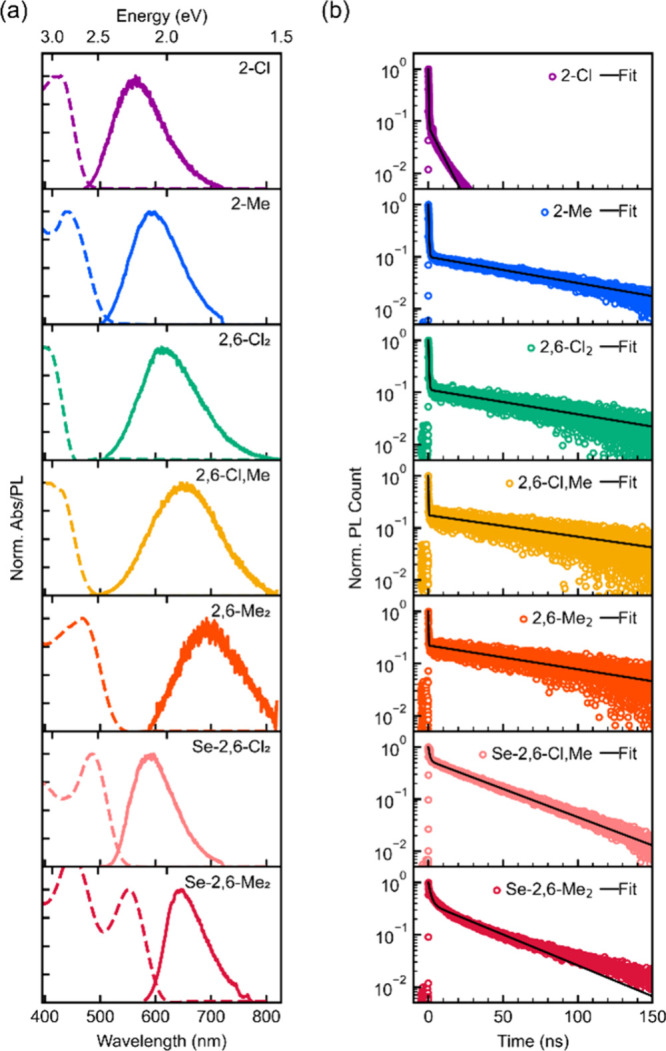
(a) Absorbance spectra (dashed lines) and PL spectra (solid lines) of 1D MOCs. (b) Time-resolved PL decay profiles of 1D MOCs with biexponential fitting curves.

**Table 2:** Optical Properties of MOCs at 300 K

MOCs	λ_abs_ (nm (eV))	λ_em_ (nm (eV))	fwhm (nm)	Stokes Shift (nm)	τ_1_/τ_2_ (ns/ns)	PLQY (%)
**2-Cl**	429 (2.89)	564 (2.20)	103	135	0.2/7.7	0.4
**2-Me**	437 (2.84)	589 (2.11)	106	152	0.3/86	8.3
**2,6-Cl**_**2**_	400 (3.10)	610 (2.03)	122	210	0.3/92	14
**2,6-Cl,Me**	428 (2.90)	644 (1.93)	143	216	0.1/105	12
**2,6-Me**_**2**_	467 (2.66)	692 (1.79)	128	225	0.1/95	6.5
**Se-2,6-Cl**_**2**_	486 (2.55)	596 (2.08)	92	110	0.9/40	8
**Se-2,6-Me**_**2**_	553 (2.24)	647 (1.92)	83	94	1.7/36	19

Among the MOCs, **2,6-Cl**_**2**_, **2,6-Cl,Me**, and **2,6-Me**_**2**_ exhibited relatively broad luminescence and large Stokes shifts (Table 2). Although the calculated band gaps of **2,6-Me**_**2**_ (Figure 3c) and **2-Me** (Figure 3g) are nearly identical at approximately 1.6 eV, the measured absorption edge of **2,6-Me**_**2**_ (Figure S10 and Table S7) was 2.42 eV, which is smaller than that of **2-Me** at 2.53 eV. The discrepancy between the calculation and experiment appears in two ways. First, the calculated band gaps are substantially smaller than the experimental absorption edges, reflecting the well-known limitation of the GGA functional, which tends to underestimate band gaps due to self-interaction error. Second, while the computed gaps for the two materials are nearly the same, their experimental absorption edges differ by 0.11 eV. This difference likely arises from stronger electron–phonon coupling in **2,6-Me**_**2**_, which can redshift the absorption onset. Such thermal effects are not captured in DFT calculations, which assume a temperature of 0 K and fixed atomic positions.

The PLQY of the MOCs ranged from 0.4 to 19% (Figure S11), comparable to recently reported 1D MOCs.^33^ For the S-based MOCs, **2,6-Cl**_**2**_ had the highest PLQY at 14% and substitution with the Me group gradually reduced the PLQY, whereas in the Se based MOCs, **Se-2,6-Me**_**2**_ exhibited a PLQY of 19%, higher than **Se-2,6-Cl**_**2**_. The moderate PLQY values observed for the MOCs may be attributed to intrinsic defects or impurities arising from variability in the quality of the synthesized crystals.

To further investigate the PL properties of the MOCs, we conducted transient PL measurements (Figure 4b). The decay profiles were well described by a biexponential function, comprising a fast prompt and a delayed decay component (Figures 4b and S12 and Table S8). Among the MOCs, **2-Cl** exhibited a faster delayed component, indicating that nonradiative decay processes dominate, which is consistent with the lower **PLQY** observed for this compound. In contrast, **2-Me** displayed bright luminescence with a longer delayed lifetime of 86 ns (Table 2). The PL decays of **2,6-Cl**_**2**_, **2,6-Cl,Me**, and **2,6-Me**_**2**_ showed similar behavior with delayed lifetimes ranging from 92 to 105 ns. In Se-containing MOCs, the delayed components decayed more quickly than those of S-based MOCs, with delayed lifetimes of approximately 40 ns. While variations in PLQY were observed among the MOCs, the emission lifetime alone cannot fully explain the results, suggesting that the materials exhibit complex exitonic behavior.

Theoretical calculations suggest that part of the synthesized MOCs are indirect band gap semiconductors, with the energy difference between the indirect and direct band gaps of less than 0.04 eV. On the other hand, experimental results reveal similar optical properties between the indirect (**2,6-Cl**_**2**_, **2,6-Cl,Me**) and the direct (**2,6-Me**_**2**_) band gap MOCs, including their absorption and PL spectra and their PL decay behavior. Further, we analyzed the absorption spectra edges using the Kubelka–Munk equation,^43^ which can distinguish between the direct and indirect band gap nature of the absorption. For all MOCs the absorption edges were found to be proportional to (*F*(*R*)*hν*)^2^, where *F*(*R*) represents the Kubelka–Munk function^43^ (Figure S10). This result suggests that with regards to the optical properties the MOC crystals behave as direct band gap semiconductors.^44^ This may also explain the higher PLQY of 14% observed for the indirect band gap MOC, **2,6-Cl**_**2**_, compared to the relatively lower PLQY of 6.5% for the direct band gap MOC, **2,6-Me**_**2**_ (Figure S11). The observed PL lifetimes, ranging from 8 to 100 ns, are consistent with those of direct band gap hybrid semiconductors, such as lead halide perovskites, as well as previously reported 1D MOCs.^28,33,45^

Next, we performed polarization-resolved PL spectroscopy on individual crystals of 1D MOCs (Figure S13). In Figure 5a, 2D polarization plots of PL intensity as a function of wavelength are presented, with **Se-2,6-Me**_**2**_ as a representative. All MOCs exhibited single emission components with a periodicity of 180°, with spectrum shape unchanged by the emission polarized angles (Figure S14). The PL intensity at the peak position was plotted in polar coordinates for each 1D MOC (Figures 5b and S14) with a fitted curve using the formula of *A* = (*A*_max_ – *A*_min_) cos^2^(θ – θ_0_) + *A*_min_, where θ_0_ denotes the reference polarization angle. All MOCs exhibit strong anisotropy along crystal orientation. The dichroism ratio of PL intensity, defined as *A*_max_/*A*_min_, ranges from 3 to 6 (Figure 5c), comparable to reported 1D hybrid semiconductors excited by circularly polarized light.^46^

**Figure 5:**
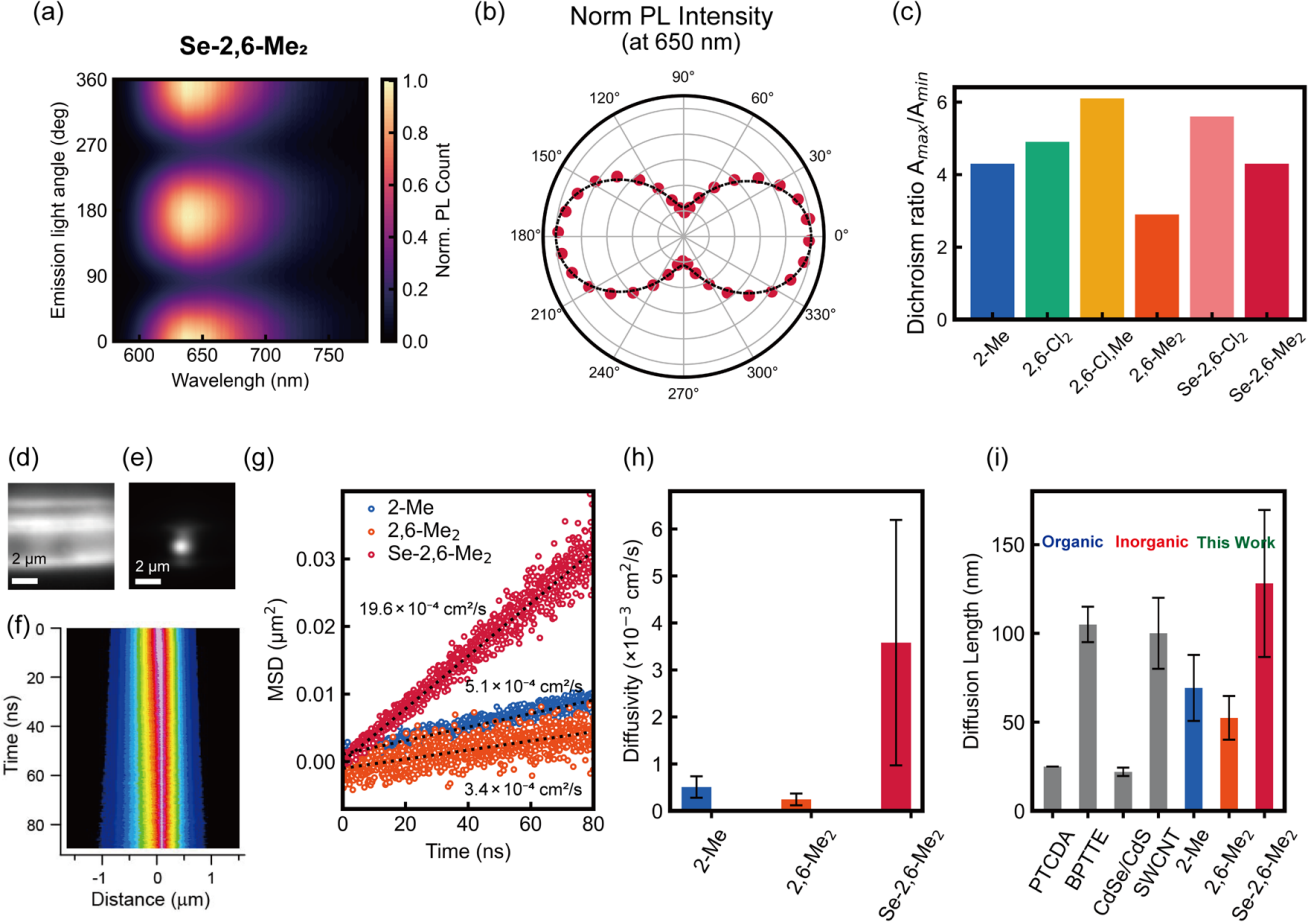
(a) 2D polarization plot of PL intensity as a function of wavelength and (b) polar plot at the PL peak (650 nm) for **Se-2,6-Me**_**2**_. Dotted lines correspond to the fitting curve. (c) Dichroism ratio of 1D MOC crystals. Image of excited **Se-2,6-Me**_**2**_ crystal by (d) wide field and (e) confocal mode. (f) Map of emission intensity in space and time as measured along the crystal orientation of **Se-2,6-Me**_**2**_ crystal. (g) Time evolution of MSD. (h) Exciton diffusivity and diffusion length along the chain of 1D MOCs for **2-Me**, **2,6-Me**_**2**_, and **Se-2,6-Me**_**2**_. (i) Comparison of exciton diffusion length with other materials (see Table S9 for the reference).

We further measured and compared exciton diffusivity of three systems: **2-Me**, **2,6-Me**_**2**_, and **Se-2,6-Me**_**2**_. Exciton diffusion provides valuable insight into the performance of emissive materials.^47^ To measure the diffusion coefficients along the crystal length, the MOC crystals were aligned horizontally. A wide-field PL microscopic image of a typical **Se-2,6-Me**_**2**_ crystal is shown in Figure 5d. The crystal was then illuminated at its center confocally with a picosecond pulsed laser with a spot size of ∼1 μm (Figure 5e). The PL intensity cross sections at the center show a uniform broadening of PL with increasing delay times (Figure 5f), indicating the presence of exciton diffusion. The diffusion analyzed in terms of mean square displacement (MSD) follows a linear trend over time, yielding a diffusion coefficient of 19.6 × 10^–4^ cm^2^/s for **Se-2,6-Me**_**2**_ (Figure 5g). Slower diffusion was observed for **2-Me** and **2,6-Me**_**2**_, with exemplar diffusion coefficients of 5.1 × 10^–4^ cm^2^/s and 3.4 × 10^–4^ cm^2^/s, respectively. We repeated the measurements at multiple locations and observed a similar trend in the diffusivity, in the ascending order of **2,6-Me**_**2**_ < **2-Me** ≪ **Se-2,6-Me**_**2**_ (Figure 5g).

The parameter relevant from a device performance perspective is a diffusion length, which is determined by PL lifetime in addition to the diffusivity. The PL lifetime is typically shorter for **Se-2,6-Me**_**2**_, compared to the other two systems which have comparable lifetimes. This leads to diffusion lengths of 52.4 ± 12.3, 69.2 ± 18.5, and 128.1 ± 41.4 nm for **2,6-Me**_**2**_, **2-Me**, and **Se-2,6-Me**_**2**_, respectively (Figure 5i). Such large exciton diffusion lengths observed in 1D MOCs are higher than most of those reported for organic semiconductors, with the exception of acene derivatives, whose diffusion lengths exceed 400 nm due to their long triplet lifetimes (Figure 5i and Table S9).^48–51^ Similarly, these values surpass those of inorganic quantum dots or 1D carbon nanotubes (Figure 5i).^52,53^

Exciton diffusion is affected by various factors, including band structure, effective masses of carriers, molecular arrangement, and defects and impurities in the crystals. Although **2-Me** was theoretically identified as an indirect band gap semiconductor, its exciton diffusion behavior closely resembles that of the direct band gap MOCs (**2,6-Me**_**2**_ and **Se-2,6-Me**_**2**_). The exciton diffusivity is strongly dependent on the crystal quality, with significant variations in local diffusive behavior observed at different locations of the same crystal. Such heterogeneity is observed predominantly in **Se-2,6-Me**_**2**_, with wide distribution of PL lifetimes and subdiffusive carrier migration at certain locations.

We also found that the exciton diffusivity correlates with the calculated effective masses of holes and electrons. DFT calculations indicate that the carrier effective masses are significantly smaller for **Se-2,6-Me**_**2**_ (*m*_e_* = 0.4, *m*_h_* = 0.2) compared to **2-Me** (*m*_e_* = 2, *m*_h_* = 0.5), and **2,6-Me**_**2**_ (*m*_e_* = 5.1, *m*_h_* = 1) (Figure S7 and Table S10). The differences in carrier effective masses provide a plausible explanation for the observed contrast in the diffusion coefficients. Future studies will focus on detailed analyses of other factors, such as defect density, crystal quality, and the morphology of the 1D chains.

Even though our primary interest is to study exciton diffusion along the crystal, we investigated the diffusion process across the crystal, as well. The exciton diffusion in the MOC crystals exhibits anisotropy, the extent of which depends on the MOC composition and location. A higher diffusion coefficient along the chain (crystal) direction is observed compared to that across the chain, with the diffusivity anisotropy ratio ranging between 2 and 5. The diffusion anisotropy is exemplified by the data for **2-Me** shown in Figure S15.

## Conclusions

In conclusion, we demonstrated the systematic ligand engineering of Ag-based 1D MOCs. Structural analysis revealed that **2,6-Cl**_**2**_, **2,6-Cl**,**Me**, and **2,6-Me**_**2**_ feature segmented Ag_4_S_4_ cores connected by S atoms, while the cores of **2-Me**, **Se-2,6-Cl**_**2**_, and **Se-2,6-Me**_**2**_ display argentophilic interactions throughout the chain. All MOCs displayed broad luminescence with their emission peaks centered between 560 and 690 nm. DFT analysis indicated that these MOCs possess characteristics of 1D semiconductors with nearly direct band gaps. The band structure varied depending on the ligands, with **2,6-Cl**_**2**_, **2,6-Cl**,**Me**, and **2,6-Me**_**2**_ exhibiting flat band structures while Se-containing MOCs displayed dispersive band structure with smaller effective carrier masses. The luminescence of MOCs was polarized along the crystal orientation. Exciton diffusion measurement along the crystal orientation revealed that the diffusivity correlates with the calculated effective masses. Among the MOCs, **Se-2,6-Me**_**2**_ showed a high exciton diffusion length of approximately 130 nm, which surpasses those of many covalent systems, including organic semiconductors. Our structural tuning approach provides a robust framework for adjusting the PL properties of 1D MOCs, enabling the design of materials with significant anisotropic characteristics and improved carrier transport properties.

## Supplementary Material



## Data Availability

All available data are included in the main article and Supporting Information.

## Notes

Experimental details, synthesis and characterization of organic precursors, additional structural and characterization data for synthesized MOCs, and theoretical calculation results (PDF)
