# Supplementary material for: 1D Silver Organochalcogenide Semiconductors: Color Tunable Luminescence, Polarized Emission, and Long-Range Exciton Diffusion
Source: J Am Chem Soc. 2025 Oct 14;147(43):39516–26. doi: 10.1021/jacs.5c12551 (PMC12670413; doi:10.1021/jacs.5c12551)
Supplement: Supplementary file 1 [file ja5c12551_si_001.pdf]

## Supporting Information for:

# 1D Silver Organochalcogenide Semiconductors: Color Tunable Luminescence, Polarized Emission, and Long-Range Exciton Diffusion

Tomoaki Sakurada,[1,2,3] Nithin Pathoor,[1] Takuma Matsumoto,[1] Rattapon Khamlue,[4] Petcharaphorn Chatsiri,[4] Jan Valenta,[5] Tadashi Kawamoto,[1] Shun Omagari,[1] William A. Tisdale,[2] Watcharaphol Paritmongkol,[4*] Yeongsu Cho,[6*] Martin Vacha[1*]

[1]Department of Materials Science and Engineering, Institute of Science Tokyo, Ookayama 2-12-1, Meguro-ku, Tokyo 152-8552, Japan

[2]Department of Chemical Engineering, Massachusetts Institute of Technology, Cambridge, Massachusetts 02139, United States

[3]Yokohama Technical Center, AGC Inc., Yokohama, Kanagawa 230-0045, Japan

[4]Department of Materials Science and Engineering, School of Molecular Science and Engineering, Vidyasirimedhi Institute of Science and Technology (VISTEC), Rayong 21210, Thailand

[5]Department of Chemical Physics and Optics, Faculty of Mathematics and Physics, Charles University, Ke Karlovu 3, 12116 Prague, Czech Republic

[6]Department of Chemistry, University of Houston, Houston, Texas 77004, United States

Email: vacha.m.aa@m.titech.ac.jp, ycho22@central.uh.edu, , watcharaphol.p@vistec.ac.th

**Table of Content**

1. Ag-based 1D MOCs ........................................................................................ 2
2. Synthesis and Characterization........................................................................ 3
3. Supplementary Figures and Tables.................................................................. 9
4. References .................................................................................................... 26

## 1. Ag-based 1D MOCs

**Table S1.** Optical Properties of Reported Ag-based MOCs.[1–7]

| Compounds | Ligand | Structure | PL peak (nm) | PLQY (%) | Ref |
|---|---|---|---|---|---|
| AgSPh-*o*-F | 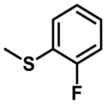 | 1D | 590 | - | 1 |
| AgSPh-*o*-$CO_2CH_3$ | 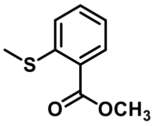 | 1D | 630 | - | 2 |
| AgSPh-*o*-$CO_2CH_3$ | 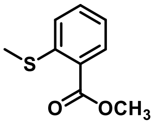 | 1D | 579 | 22 | 3 |
| AgSPh-*o*-$CO_2H$ | 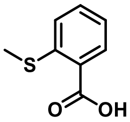 | 1D | 634 | 52 | 4 |
| AgSPh-*o*-$OCH_3$ | 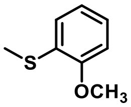 | 1D | 555 | ~0 | 3 |
| AgSPh-*m*-$CO_2CH_3$ | 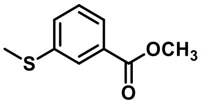 | 1D | 610 | - | 5 |
| AgSPh-*m*-$OCH_3$ | 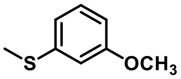 | 1D | 589 | ~1 | 3 |
| AgSPh-*m*-$CO_2H$ | 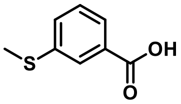 | 2D | 460, 555 | - | 5 |
| AgSPh-*p*-$CO_2CH_3$ | 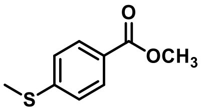 | 2D | 489, 650[a] | - | 6 |
| AgSPh-*p*-$CO_2CH_3$ | 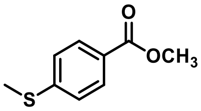 | 2D | - | - | 3 |
| AgSPh-*p*-$CO_2H$ | 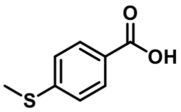 | 2D | 484, 700[a] | - | 6 |
| AgSPh-*p*-$OCH_3$ | 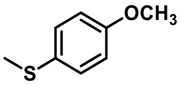 | 2D | - | - | 3 |
| AgSePh-*o*-$F_2$(2,6) | 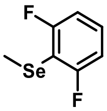 | 1D | 574 | 2.4 | 7 |

a: Luminescence at 93 K.

## 2. Synthesis and Characterization

### 2.1. Chemicals

Solvents and reagents were purchased from TCI, MilliporeSigma, or Fisher Scientific and used without any further purification.

### 2.2. Synthesis

#### 2.2.1. Synthesis of Diselenides

*Synthesis of 1,2-bis(2,6-dichlorophenyl) diselenide (**1**)*

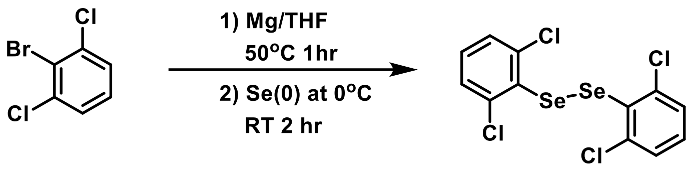


To a mixture of Mg (2.52 g, 110 mmol) and 100 mL of anhydrous tetrahydrofuran (THF), 1-bromo-2,6-dichlorobenzene (22.6 g, 100 mmol) was added and stirred under $N_2$ atmosphere for 20 min. The reaction mixture was heated and stirred at 50 °C for 1 h before being cooled in an ice bath. Elemental selenium (8.0 g, 100 mmol) was added in a single portion, and the reaction mixture was allowed to warm to room temperature and stirred for 1 h. The mixture was then filtered thorough Celite and left at ambient condition overnight. After that, the solvent was evaporated, and the obtained residue was redissolved in dichloromethane. The organic phase was washed with an aqueous ammonium chloride solution and brine, then dried over $Na_2SO_4$. Solvent was removed under reduced pressure using a rotary evaporator, and the crude product was purified by column chromatography using hexanes as the eluent. Diselenides were obtained as an orange oil (*ca.* 10g), which later transformed into yellow crystals when stored at ambient conditions for 1 week (6.5 g, 29% yield).

$^1$H-NMR (400 MHz, $CDCl_3$) $\delta$ 7.35-7.33 (m, 4H), 7.18 (dd, $J$ = 8.5, 7.5 Hz, 2H). $^{13}$C-NMR (101 MHz, $CDCl_3$) $\delta$ 142.0, 131.2, 128.1.

*Synthesis of 1,2-bis(2,6-dimethylphenyl) diselenide (**2**)*

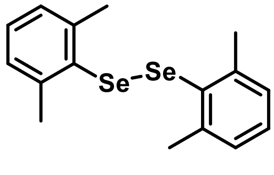


Orange crystals (40% yield)

$^1$H-NMR (400 MHz, $CDCl_3$) $\delta$ 7.08 (dd, $J$ = 8.3, 6.4 Hz, 2H), 7.01-6.99 (m, 4H), 2.26 (s, 12H). $^{13}$C-NMR (101 MHz, $CDCl_3$) $\delta$ 144.0, 132.1, 129.4, 127.6, 24.4.

**Table S2**. Crystallographic data for diselenides

| | (**1**) | (**2**) |
|---|---|---|
| CCDC | 2464833 | 2464831 |
| Empirical formula | $C_{12}H_6Cl_4Se_2$ | $C_{16}H_{18}Se_2$ |
| $M_r$ | 449.89 | 368.22 |
| Temperature (K) | 113(2) | 113(2) |
| Wavelength (Å) | 0.71073 | 0.71073 |
| Crystal system | Triclinic | Monoclinic |
| Space group | $P$-1 | $P2_1/n$ |
| $a$ (Å) | 7.8456(3) | 9.4607(4) |
| $b$ (Å) | 7.8886(3) | 13.6634(6) |
| $c$ (Å) | 11.2747(4) | 11.2006(5) |
| α (°) | 78.982(3) | 90 |
| β (°) | 86.620(3) | 96.036(4) |
| γ (°) | 84.566(3) | 90 |
| $V$ (Å$^3$) | 681.27(5) | 1439.83(10) |
| $Z$ | 2 | 4 |
| Calculated density (Mg/m$^3$) | 2.193 | 1.699 |
| Absorption coefficient (mm$^{-1}$) | 6.187 | 5.116 |
| $F$(000) | 428 | 728 |
| Crystal size (mm$^3$) | 0.40 × 0.40 × 0.20 | 0.30 × 0.30 × 0.20 |
| $\theta$ range for data collection (°) | 2.610 to 30.561 | 2.628 to 30.590 |
| Index ranges | $-11 \leq h \leq 11$, $-11 \leq k \leq 11$, $-16 \leq l \leq 15$ | $-13 \leq h \leq 13$, $-19 \leq k \leq 18$, $-14 \leq l \leq 15$ |
| Reflections collected | 10182 | 11965 |
| Independent reflections | 3926 [$R_{int}$ = 0.1282] | 4248 [$R_{int}$ = 0.1383] |
| Completeness to $\theta$ = 25.242° | 99.4% | 99.9% |
| Data / restraints / parameters | 3926 / 0 / 164 | 4248 / 0 / 168 |
| Goodness-of-fit on $F^2$ | 1.005 | 1.029 |
| Final $R$ indices [$I > 2\sigma(I)$] | $R1$ = 0.0549, $wR2$ = 0.1446 | $R1$ = 0.0529, $wR2$ = 0.1356 |
| $R$ indices (all data) | $R1$ = 0.0630, $wR2$ = 0.1531 | $R1$ = 0.0648, $wR2$ = 0.1471 |
| Largest diff. peak and hole (e.Å$^{-3}$) | 1.325 and −1.670 | 1.571 and −1.206 |

### 2.2.2. Synthesis of MOC crystals[8]

A 100 mM solution of $AgNO_3$ in 1-butylamine (10 mL) was mixed with a 100 mM solution of organothiol in 1-butylamine (10 mL) and filtered through 0.2 µm PTFE filter. A 30 mL glass vial containing 20 mL of filtered solution was placed into a 110 mL glass vial containing deionized water (DI, 20 mL), allowing water to diffuse into the vial and organic solvents to evaporate. After 6 days, crystals appeared. Crystals were filtered and washed with methanol and dried under vacuum.

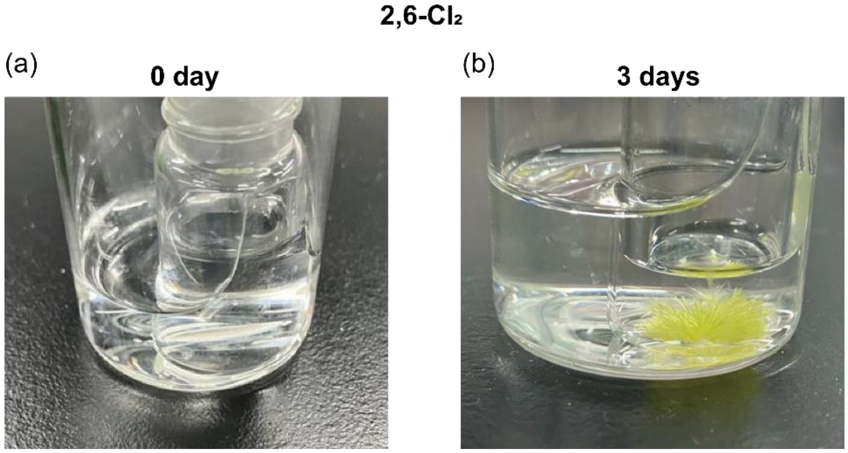


**Figure S1.** Optical images of the reaction vial placed in a jar containing antisolvent: (a) after mixing the solutions and (b) after 3 days of crystal growth.

### 2.2.3. Recrystallization of MOC (2,6-Me$_2$, 2,6-Cl$_2$, Se-2,6-Me$_2$, and Se-2,6-Cl$_2$)

MOC microcrystalline powders were dissolved in diaminopropane with the concentration of ~3 mg/mL, then filtered through a 0.22 µm syringe filter to obtain a clear solution. A 1 mL aliquot of the filtered solution was transferred into a 4 mL vial, which was then placed inside a capped 20 mL vial containing 3 mL of a different antisolvent (Ethanol, DI, Toluene or BuOH). Crystals formed over 3 - 14 days. For structural determination by SCXRD, the crystals were directly collected from the solution without further purification.

### 2.3. Characterization

#### 2.3.1. Nuclear magnetic resonance (NMR) measurement

NMR spectra were recorded with a Bruker DMX 400 MHz spectrometer at 298 K. The spectral data are reported as chemical shift (in ppm). Chemical shifts were calibrated against peak of a reference chemical as internal standard ($^{1}$H-NMR: Tetramethylsilane $\delta$ = 0 ppm) or a solvent ($^{13}$C-NMR: Chloroform $\delta$ = 77.16 ppm).

#### 2.3.2. Thermogravimetric analysis (TGA)

TGA data was collected with a TGA Q500 differential thermal analyzer. The samples were heated from room temperature to 500 °C with a heating rate of 5°C min$^{-1}$ under $N_2$ stream (40 mL min$^{-1}$).

#### 2.3.3. Powder X-ray Diffraction (PXRD)

Powder X-ray diffraction data were collected using a Rigaku SmartLab X-ray diffractometer (Cu $K_\alpha$ radiation, $\lambda$ = 1.54184 Å). A 0.04 rad Soller slit, a 2° antiscatter slit, a 10 mm mask, and a programmable divergence slit with an illuminated length of 6 mm were used in the incident beam path. The diffracted beam optics included a 0.04 rad Soller slit, a Ni filter, and an automatic receiving slit.

#### 2.3.4. Scanning Electron Microscopy (SEM)

SEM images were obtained using a KEYENCE 3D microscope VE-9800 with an accelerating voltage of 15 kV. High-magnification and low-magnification images were captured at 3000× and 100×, respectively.

#### 2.3.5. UV-Vis Diffuse Reflectance Spectroscopy

Measurements of diffuse reflectance were performed on a PerkinElmer Lambda 1050 UV-vis-NIR spectrometer equipped with a universal reflectance accessory. Solid samples were prepared by grinding with dry potassium bromide (KBr) to a ∼1 wt% dilution and diffuse reflectance spectra were normalized to a 100% KBr baseline. The obtained diffuse reflectance spectra were converted into absorption spectra by Kubelka−Munk transform[9]:

$$F(R) = \frac{(1-R)^2}{2R}$$

where $F(R)$ is the Kubelka−Munk[9] function with a value proportional to the sample's absorption coefficient, and $R$ is the relative reflectance of the sample with the 100% KBr baseline.

### 2.3.6. Single-Crystal X-ray Diffraction

*For organodiselenides (**1**), (**2**), and **2-Me***

X-ray diffraction data were collected using a using XtaLAB Mini II (Rigaku) diffractometer using Mo Kα radiation (λ = 0.71073 Å). The crystal was kept at a steady $T$ = 113.15 K during data collection. The structure was solved with the ShelXT[10] structure solution program using the Intrinsic Phasing solution method and by using Olex2[11] as the graphical interface. The model was refined with version 2018/3 of ShelXL 2018/3 using Least Squares minimisation.[12]

Compound **1** crystallizes in the triclinic centrosymmetric space group $P$-1, and compound **2** crystallizes in the monoclinic space group $P2_1/n$. Compound **2-Me** crystallized in the triclinic centrosymmetric space group $P$-1. Compound **2,6-Me2** crystallizes in the monoclinic space group $P2/c$.

*For **2,6-Cl$_2$**, **2,6-Cl,Me**, and **2,6-Me$_2$**, **Se-2,6-Cl$_2$**, and **Se-2,6-Me$_2$***[13]

X-ray diffraction data were collected using a Bruker-AXS D8 Venture diffractometer equipped with a IμS micro-source and a Photon 3 CPAD detector, using Mo Kα radiation (λ = 0.71073 Å). The crystal was kept at a steady $T$ = 100.00 K during data collection. The crystal structures were processed in APEX5 and solved in OLEX2 program[11] using dual-space methods with SHELXT[10] and refined against $F^2$ using full-matrix least squares with SHELXL-2017.[12] All non-hydrogen atoms were refined anisotropically, while hydrogen atoms were placed at geometrically calculated positions and refined using a riding model. For **2,6-Cl,Me**, the Chlorine and Carbon atom at *ortho* position were refined by constraining these two atom coordinates (EXYZ) of to be equal.

### 2.3.7. Polarization resolved PL micro-spectroscopy

Steady-state PL measurements and polarized emission measurements were performed on an inverted microscope (Olympus, IX71) under ambient conditions . The samples were excited by focusing the output of a 458 nm laser diode (Fianium, WL-SC) using an objective lens (Olympus, UPLanFL, 100×, 1.30 NA) to a ~1 μm spot. The excitation light was converted to circularly polarized light by a λ/4 plate. After excitation, the PL was collected in the epi-configuration and passed through a dichroic mirror and a long-pass filter. It was then directed into a CCD detector (Andor Technology, EM-CCD, iXon). For the emission polarization measurements, a linear polarizer (Sigma Koki, SPF-50C-32) was placed between the dichroic mirror and the CCD detector, and was mechanically controlled by a rotator (Thorlabs, KPRM1E and KDC101). Optical measurement setup is shown in **Figure S12**. For PL spectroscopic measurements the emission was directed to the CCD via a monochromator.

### 2.3.8. Measurement of PL lifetime, exciton diffusion coefficient and diffusion lengths

The diffusion of excited carriers was measured with a home-built setup based on a widefield epi-fluorescence microscope (Olympus, IX73). The excitation path is modified for interchangeable widefield-confocal excitation, and emission path with spatially resolved lifetime measurements. We used a picosecond-pulsed 375 nm laser (PicoQuant, LDH-D-C375 laser controlled via PDL 800-D), and illuminated the sample with 10 MHz repetition rate via an oil immersion objective (Olympus, 1.3 NA, 100×). The PL emission is collected via the same objective lens. A dichroic beamsplitter (Semrock, FF376-Di01-25×36) and an emission filter (Semrock, FF01-380/LP-25) was used for laser illumination and isolation of emitted light from the sample. The fwhm of the confocal spot was measured as ~400 nm with a laser power of ~0.9 fJ/pulse, measured after the objective lens. For PL imaging, the emission was collected with an EMCCD (Andor iXon3) through a pair of riley lenses ($f$ =70 mm). For diffusivity measurement, the emission was diverted into an avalanche photodiode, APD (Micro Photon Devices SPD-050-CTC) with a flipper mirror between the riley lenses. A long focal length ($f$ = 300 mm) lens is used to form an image with ~430x magnification in APD detection plane. The APD with 50 μm × 50 μm active area (equivalent to ~115 nm × 115 nm) is placed on a 1D piezo-stage and scanned across the confocal spot at a step-size of 30 μm (equivalent to ~70 nm steps). PL lifetime was collected at each location with 16 ps time binning using HydraHarp 400 multi-channel picosecond event timer from PicoQuant.

MATLAB code is written for the data analysis and calculation of diffusion coefficient. The spatially resolved lifetime measurement is used to generate 2D map of PL intensity cross-section evolution over delay time. The intensity cross-sections are fitted with a Gaussian function after binned to 80 ps, extracted variance to calculate Mean Square Displacement with the equation, $MSD = \sigma(t)^2 - \sigma(0)^2 = 2Dt^{\alpha}$. The diffusion length is calculated with the formula, $L_D = \sqrt{D\tau}$, where t is PL lifetime.

### 2.3.9. Density functional theory Calculations

Density functional theory (DFT) calculations were performed using Vienna Ab initio Simulation Package (VASP).[14–17] Perdew-Burke-Ernzerhof (PBE) exchange-correlation functional[18] was employed along with projector-augmented wave (PAW) pseudopotential.[19] A kinetic energy cutoff of 1000 eV was applied, and dispersion interactions were included using the DFT-D3 correction[20] with the Becke-Johnson damping.[21] A 2×2×6 k-point mesh was used for 2,6-$Cl_2$ and 2,6-Cl,Me, a 2×6×2 mesh for 2,6-$Me_2$, and a 6×2×2 mesh for 2-Me, Se-2,6-$Cl_2$, and Se-2,6-$Me_2$. The effective masses were estimated by approximating the band edge using the Kane quasi-linear dispersion relation,[22,23] as implemented in the Effmass package.[24]

## 3. Supplementary Figures and Tables

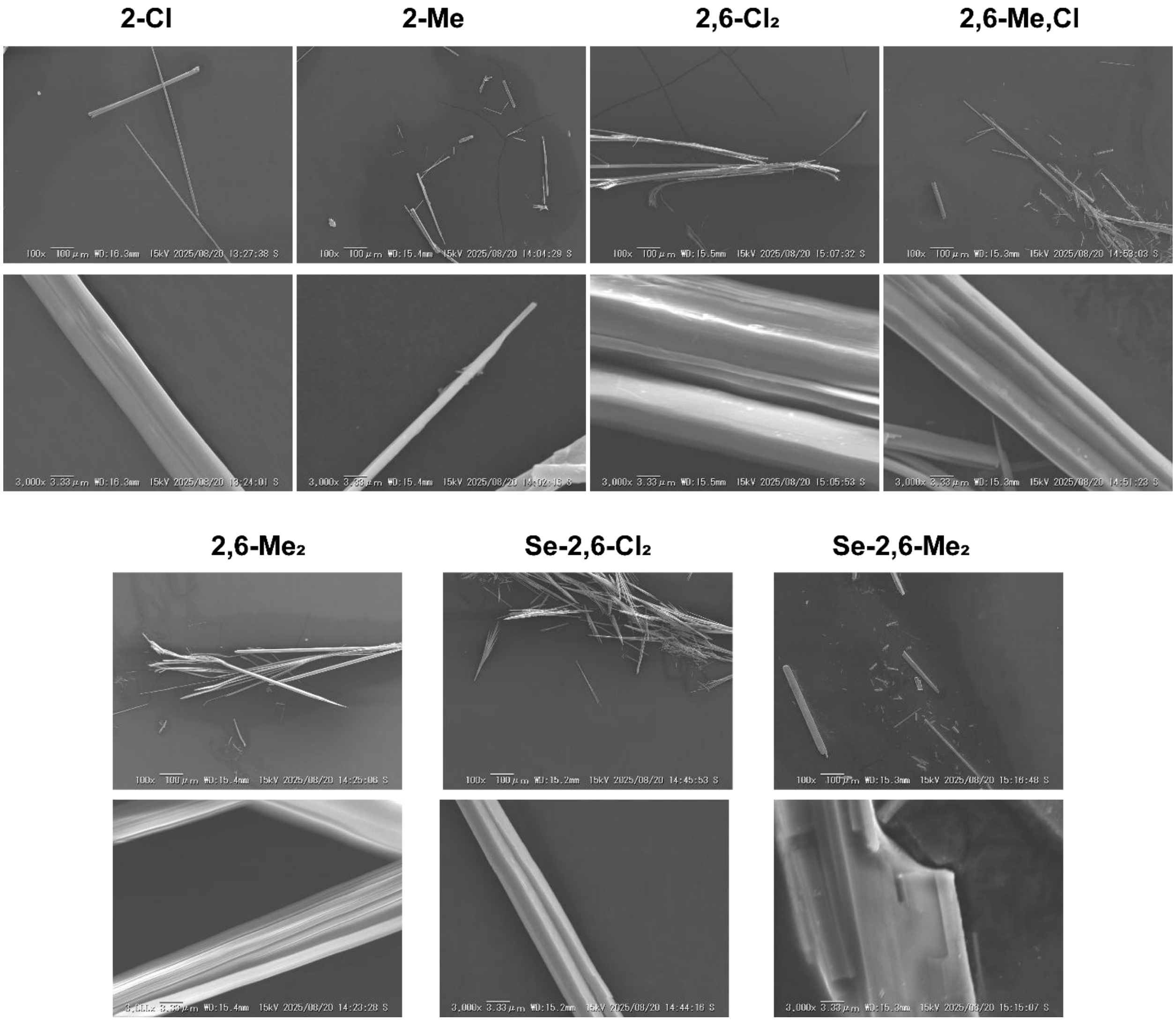


**Figure S2.** SEM images of MOC crystals at low magnification (top) and high magnification (bottom). The scale bars represent 100 μm for low magnification and 3.33 μm for high magnification.

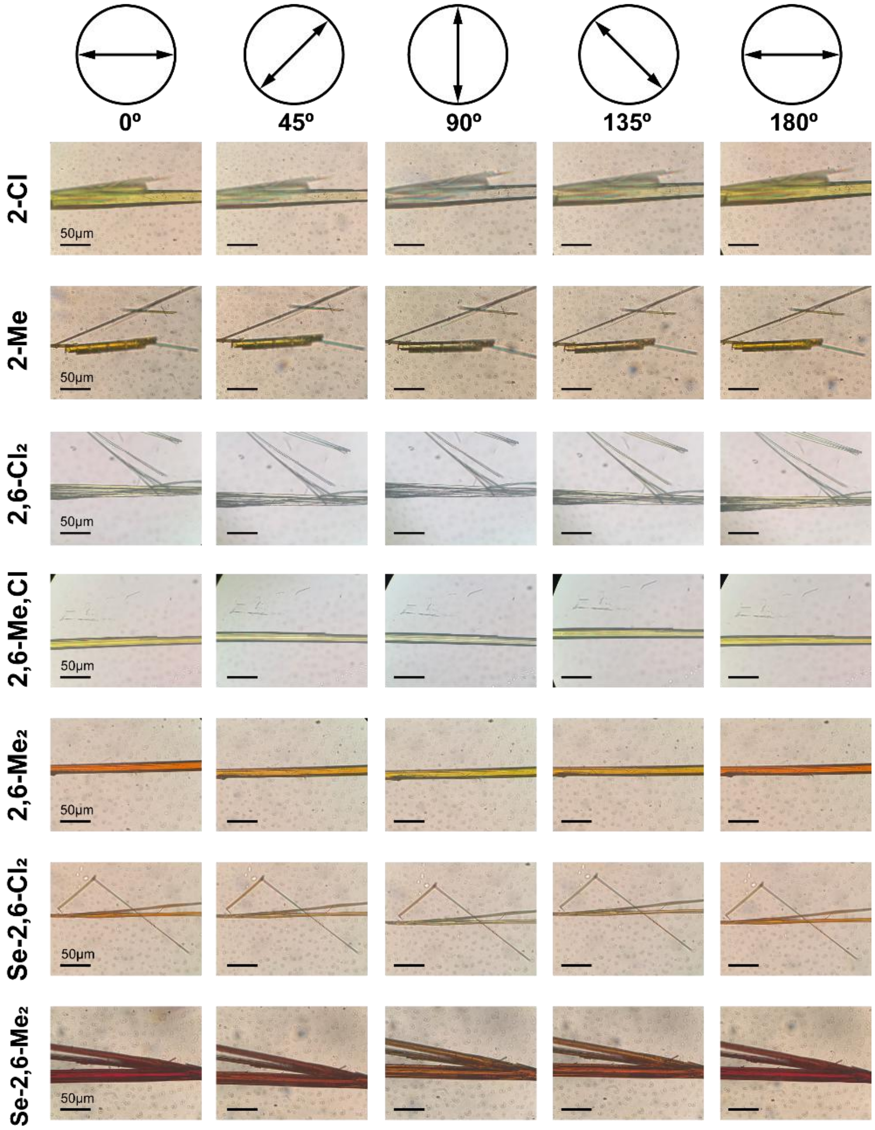


**Figure S3.** Optical images of single crystals of MOCs observed under polarized light at different polarization angles ranging from 0º to 180º.

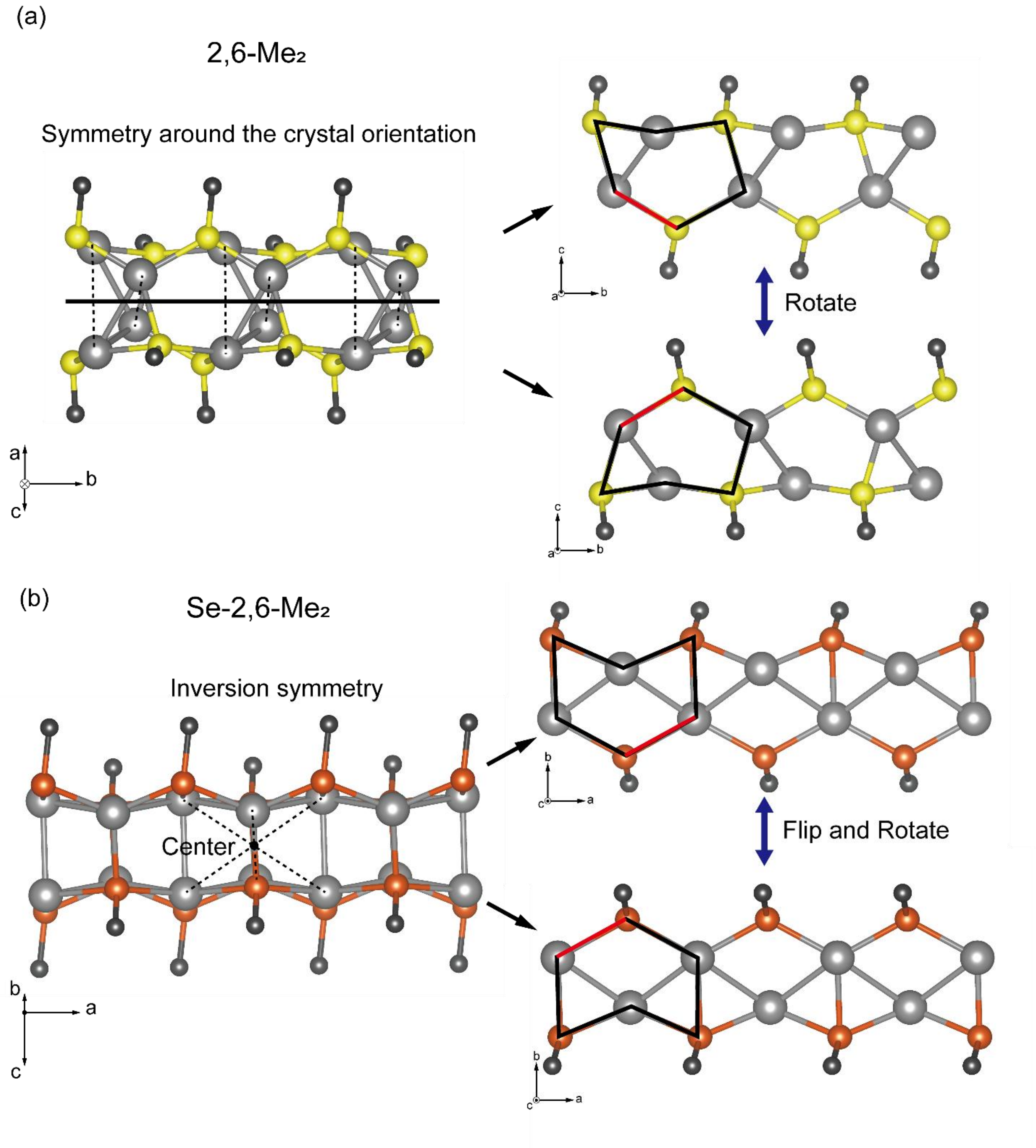


**Figure S4.** Structures of Ag-chalcogen cores of (a) **2,6-Me$_2$** and (b) **Se-2,6-Me$_2$**. The core of **2,6-Me$_2$** exhibits symmetry around the crystal orientation axis (a, left) with two identical Ag-S layers rotated around crystal orientation axis and facing each other (a, right). The cores of **Se-2,6-Me$_2$** exhibits inversion symmetry (b, left) with two identical Ag-Se layers flipped and rotated to face each other (b, right).

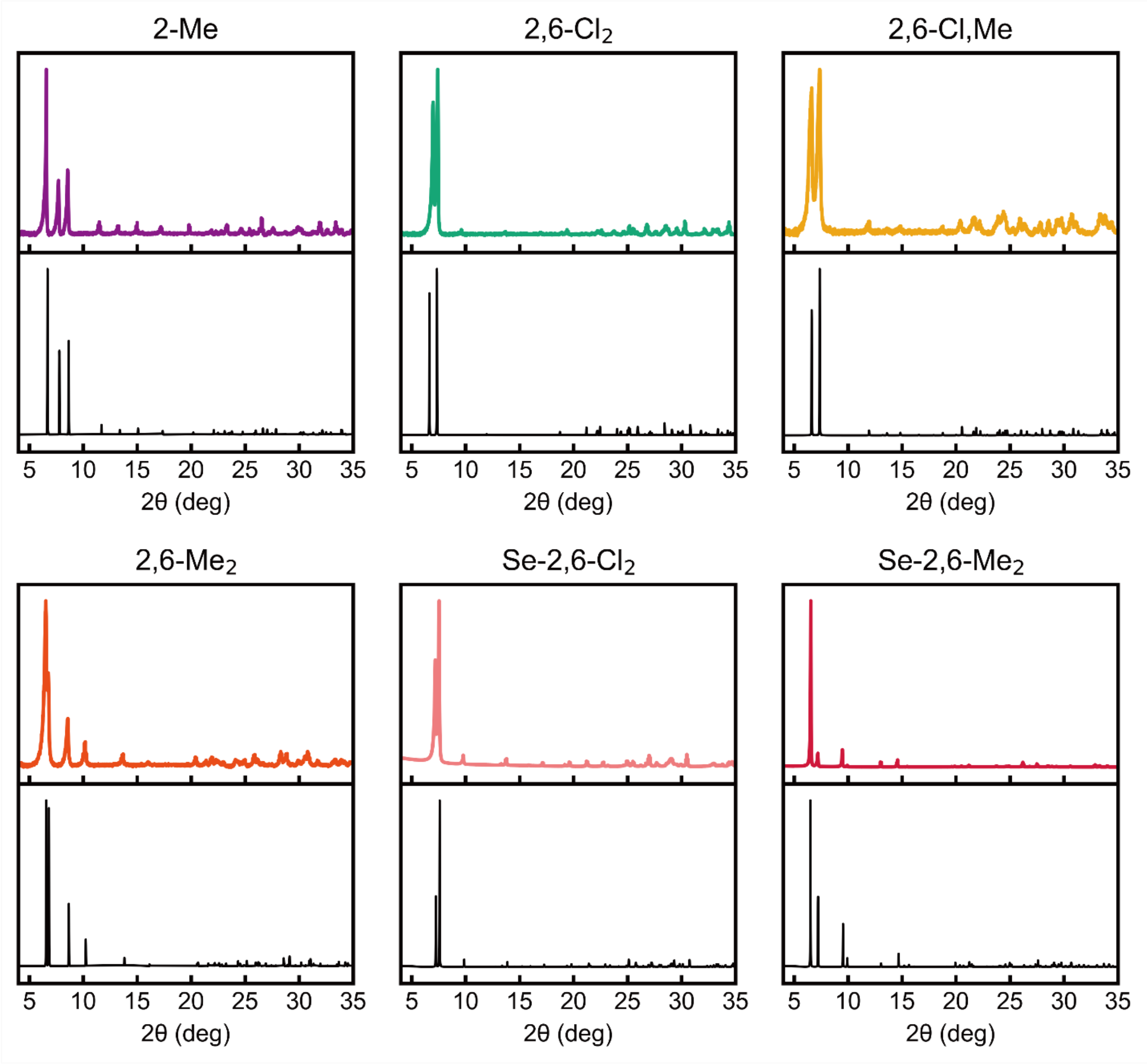


**Figure S5.** PXRD patterns of ground crystals of **2-Me**, **2,6-Cl$_2$**, **2,6-Cl,Me**, **2,6-Me$_2$**, **Se-2,6-Cl$_2$**, and **Se-2,6-Me$_2$**, along with the simulated pattern (black).

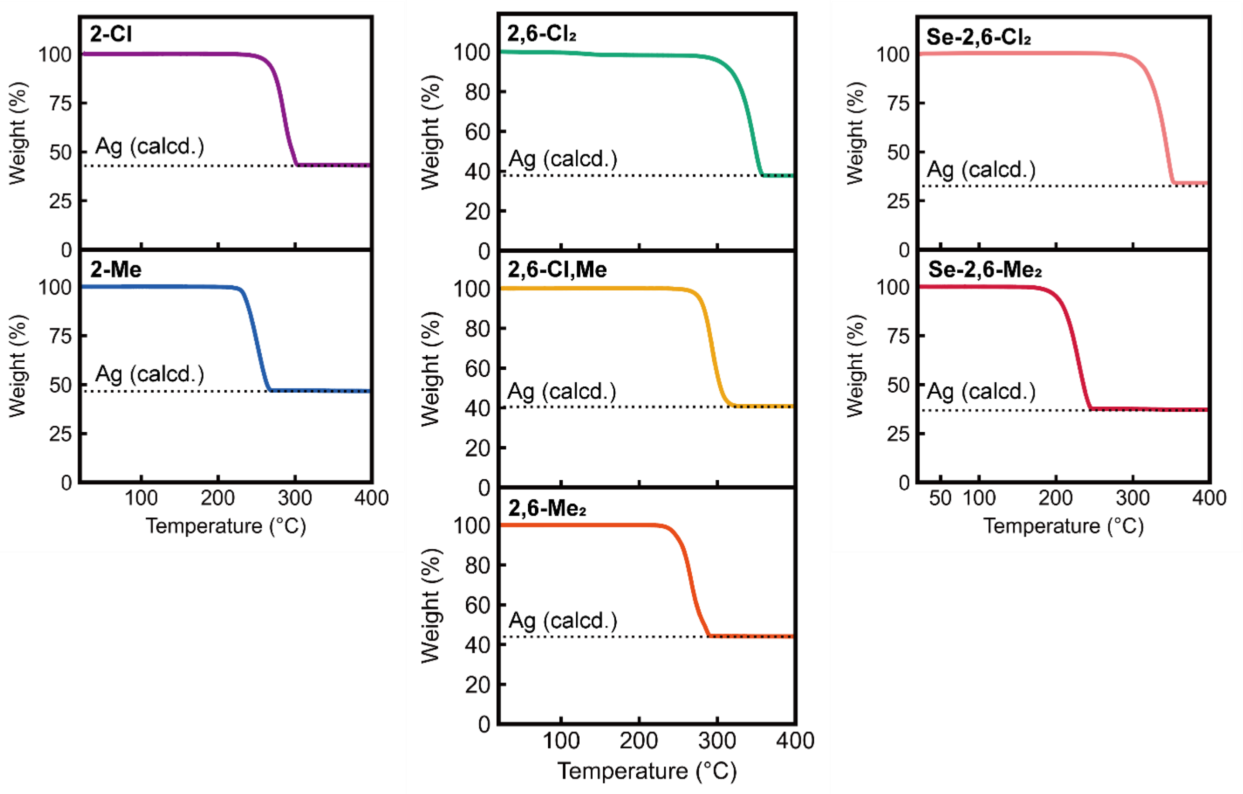


**Figure S6.** TGA curves of **2-Cl**, **2-Me**, **2,6-Cl$_2$**, **2,6-Cl,Me**, **2,6-Me$_2$**, **Se-2,6-Cl$_2$**, and **Se-2,6-Me$_2$** measured under $N_2$ atmosphere. Dotted lines represent the calculated silver content in each MOC.

**Table S3.** Decompositon temperature of MOCs and residual weights at 400 °C

| | Decomposition temperature at weight loss of 5% and 10% [°C] | | Residual weight [wt%] | Calculated Ag content [wt%] |
|---|---|---|---|---|
| | 5% | 10% | | |
| **2-Cl** | 266 | 273 | 43.0 | 42.9 |
| **2-Me** | 235 | 239 | 46.7 | 46.7 |
| **2,6-Cl$_2$** | 303 | 318 | 37.6 | 37.7 |
| **2,6-Cl,Me**, | 277 | 283 | 40.7 | 40.6 |
| **2,6-Me$_2$** | 247 | 254 | 44.0 | 44.0 |
| **Se-2,6-Cl$_2$** | 310 | 319 | 33.9 | 32.4 |
| **Se-2,6-Me$_2$** | 200 | 209 | 37.2 | 37.0 |

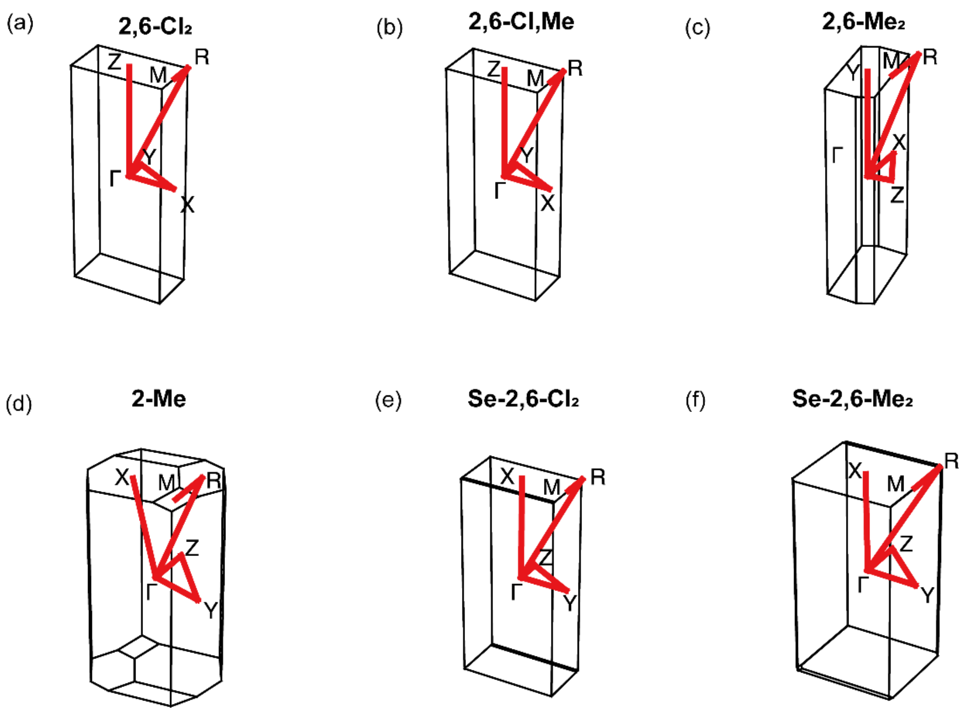


**Figure S7.** The first Brillouin zone of (a) **2,6-Cl$_2$**, (b) **2,6-Cl,Me**, (c) **2,6-Me$_2$**, (d) **2-Me**, (e) **Se-2,6-Cl$_2$**, and (f) **Se-2,6-Me$_2$**. The k-path chosen for the band structure plot is indicated by red lines.

**Table S4.** k-point coordinates in units of reciprocal lattice vectors at the valence band maximum (VBM) and the conduction band minimum (CBM), along with the direct and indirect band gaps, and the bandwidth of the lowest conduction band (CB).

| | **2,6-Cl$_2$** | **2,6-Cl,Me** | **2,6-Me$_2$** |
|---|---|---|---|
| k at VBM | (0.00, 0.00, 0.06) | (0.00, 0.00, 0.06) | (0.50, 0.00, 0.00) |
| k at CBM | (0.50, 0.00, 0.00) | (0.44, 0.00, 0.00) | (0.50, 0.00, 0.00) |
| Direct gap (eV) | 2.13 | 1.96 | 1.63 |
| Indirect gap (eV) | 2.09 | 1.94 | - |
| Bandwidth of CB (eV) | 0.29 | 0.40 | 0.44 |
| | **2-Me** | **Se-2,6-Cl$_2$** | **Se-2,6-Me$_2$** |
| k at VBM | (0.00, 0.38, 0.13) | (0.00, 0.50, 0.00) | (0.00, 0.00, 0.50) |
| k at CBM | (0.00, 0.13, 0.38) | (0.00, 0.50, 0.00) | (0.00, 0.00, 0.50) |
| Direct gap (eV) | 1.65 | 1.08 | 0.94 |
| Indirect gap (eV) | 1.64 | - | - |
| Bandwidth of CB (eV) | 0.58 | 1.02 | 1.22 |

**Table S5.** Normalized orbital contributions to the VBM, calculated using DFT with the PBE functional.

| | **2-Me** | **2,6-Me$_2$** | **2,6-Cl,Me** | **2,6-Cl$_2$** | **Se-2,6-Me$_2$** | **Se-2,6-Cl$_2$** |
|---|---|---|---|---|---|---|
| Ag 4d | 0.37 | 0.37 | 0.40 | 0.40 | 0.41 | 0.44 |
| Ag 5p | 0.04 | 0.06 | 0.05 | 0.03 | 0.04 | 0.04 |
| S 3p/Se 4p | 0.43 | 0.46 | 0.46 | 0.44 | 0.47 | 0.45 |
| C 2p | 0.15 | 0.11 | 0.08 | 0.07 | 0.08 | 0.07 |

**Table S6.** Normalized orbital contributions to the CBM, calculated using DFT with the PBE functional.

| | **2-Me** | **2,6-Me$_2$** | **2,6-Cl,Me** | **2,6-Cl$_2$** | **Se-2,6-Me$_2$** | **Se-2,6-Cl$_2$** |
|---|---|---|---|---|---|---|
| Ag 4d | 0.18 | 0.25 | 0.23 | 0.21 | 0.11 | 0.10 |
| Ag 5p | 0.05 | 0.07 | 0.07 | 0.07 | 0.06 | 0.05 |
| Ag 5s | 0.24 | 0.16 | 0.13 | 0.09 | 0.29 | 0.26 |
| S 3p/Se 4p | 0.22 | 0.20 | 0.13 | 0.09 | 0.19 | 0.15 |
| S 3s/Se 4s | 0.15 | 0.15 | 0.14 | 0.12 | 0.17 | 0.19 |
| C 2p | 0.14 | 0.15 | 0.26 | 0.36 | 0.14 | 0.15 |

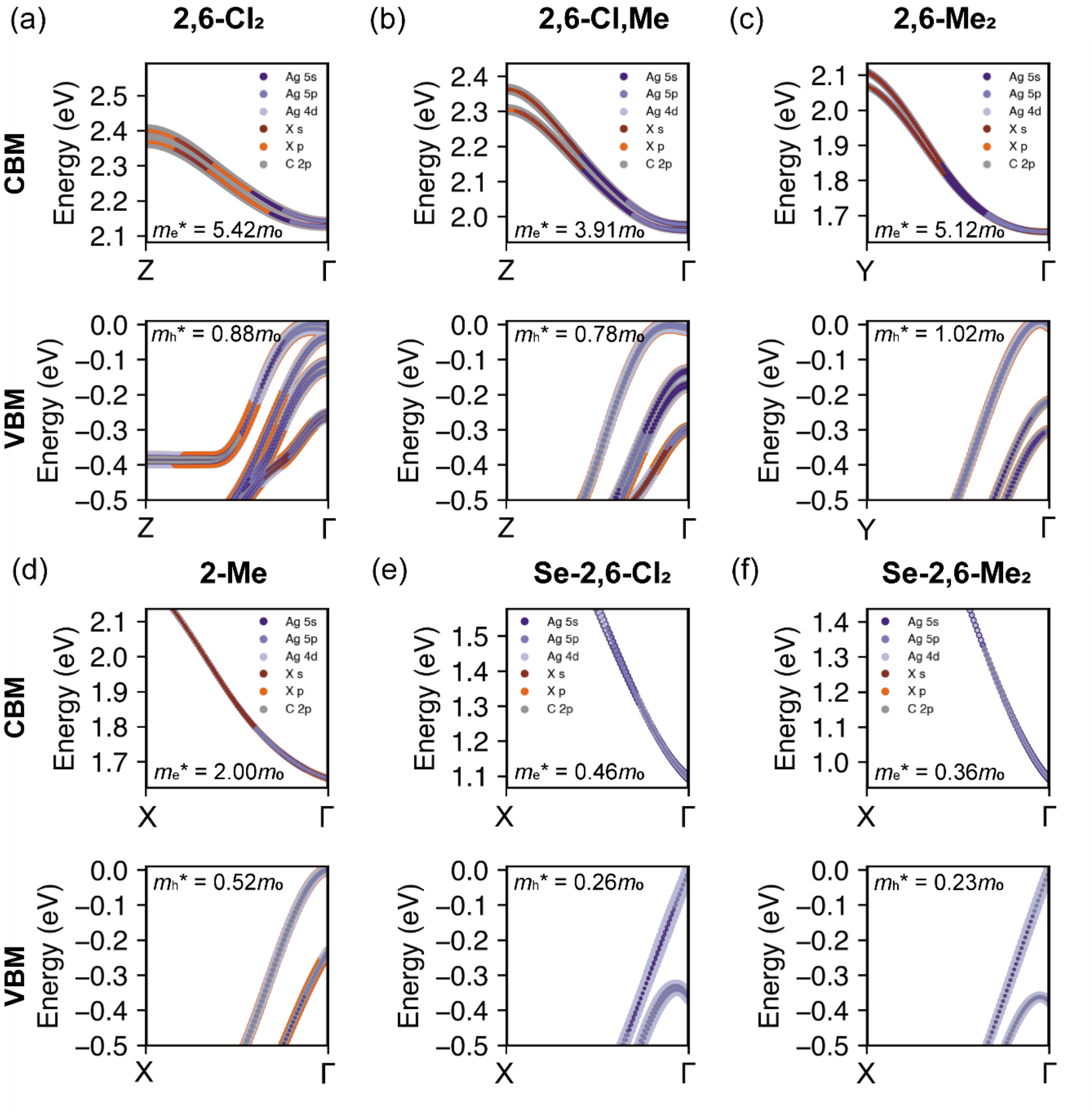


**Figure S8.** Electronic band structure of (a) **2,6-Cl$_2$**, (b) **2,6-Cl,Me**, (c) **2,6-Me$_2$**, (d) **2-Me**, (e) **Se-2,6-Cl$_2$**, and (f) **Se-2,6-Me$_2$** zoomed to the band edge of the valence band and the conduction band. The band thickness represents the contribution from each orbital.

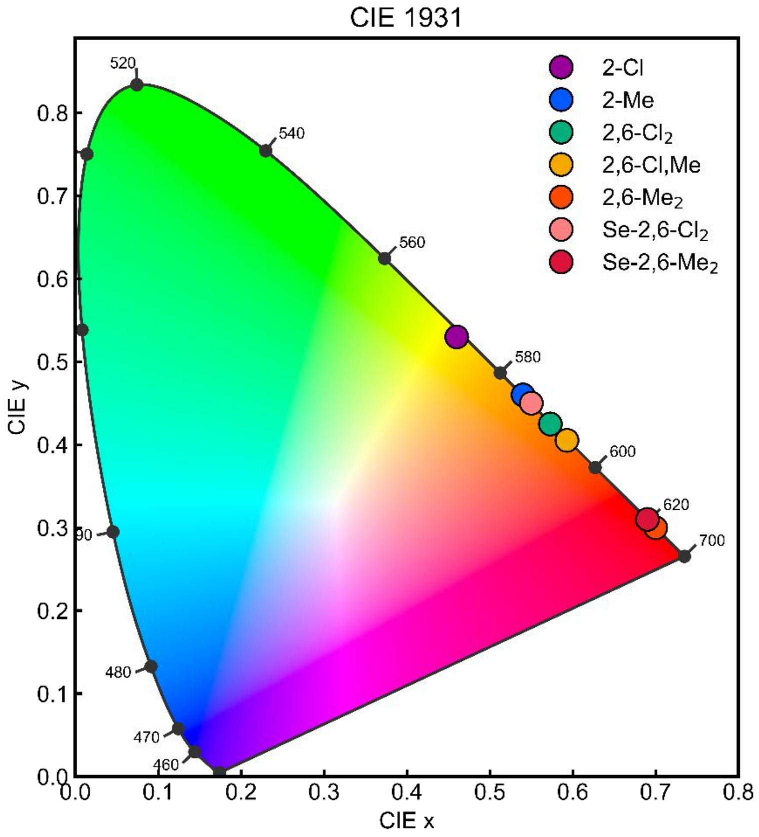


**Figure S9.** CIE 1931 [x, y] coordinates of synthesized MOCs.

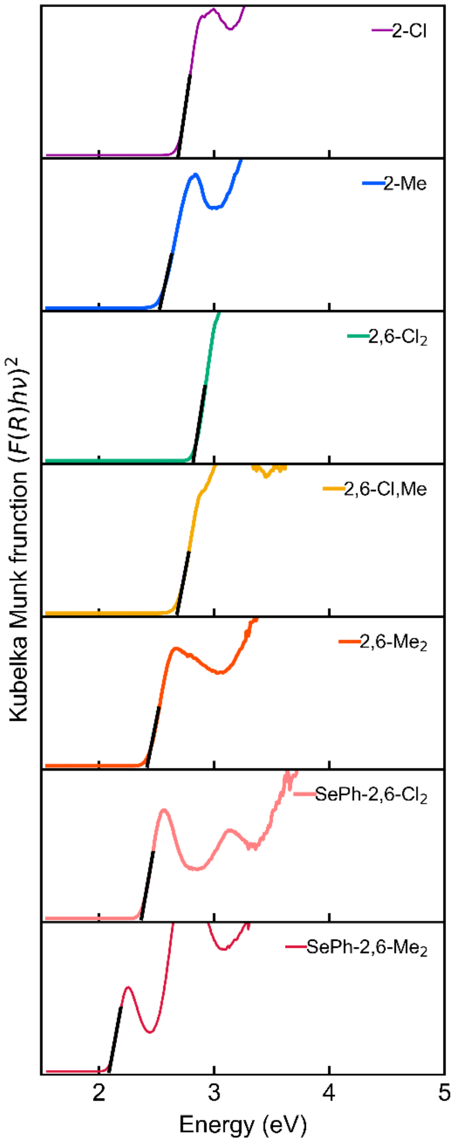


**Figure S10.** $(F(R)h\nu)^2$ plots as a function of photon energy.

**Table S7.** Calculated and experimenntally obtained band gaps of MOCs.

| MOCs | Calculated band gap (eV) | Experimental band gap (eV) |
|---|---|---|
| **2-Me** | 1.64 | 2.53 |
| **2,6-Cl$_2$** | 2.11 | 2.82 |
| **2,6-Cl,Me** | 1.94 | 2.68 |
| **2,6-Me$_2$** | 1.63 | 2.42 |
| **Se-2,6-Cl$_2$** | 1.08 | 2.37 |
| **Se-2,6-Me$_2$** | 0.94 | 2.09 |

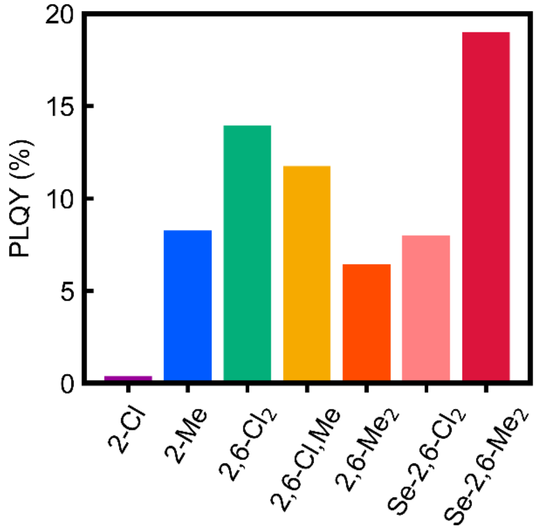


**Figure S11.** PLQY of synthesized MOCs.

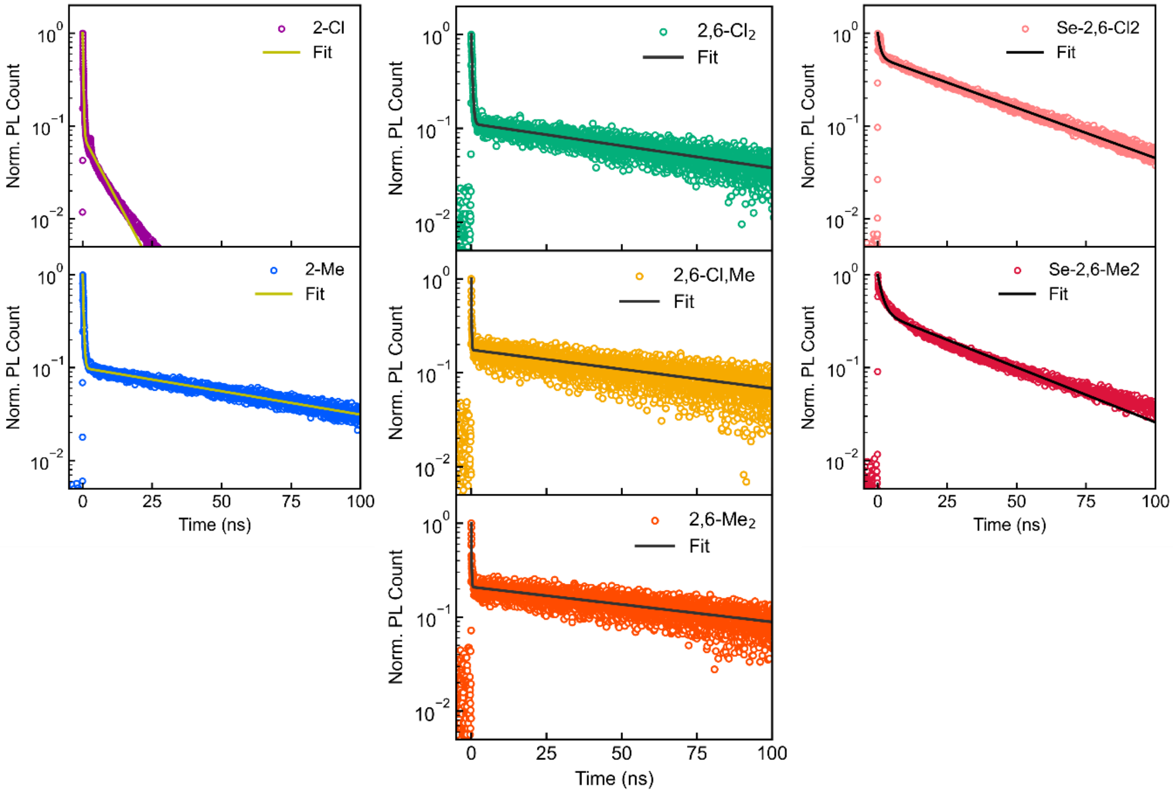


**Figure S12.** TRPL decay profiles of MOCs along with bi-exponential fitting curves.

Bi-exponential decay fitting equation for PL intensity.

$$I(t) = (1 - a)\exp\left(-\frac{t}{\tau_1}\right) + a\exp\left(-\frac{t}{\tau_2}\right)$$

Photo count calculation for two decay components

$$\int_0^{\infty} I(t)dt = \int_0^{\infty} \left\{(1 - a)\exp\left(-\frac{t}{\tau_1}\right) + a\exp\left(-\frac{t}{\tau_2}\right)\right\} dt$$

$$= (1 - a)\tau_1 + a\tau_2$$

**Table S8.** Calculated decay components and PL count ratio

| MOCs | Prompt $\tau_1$ (ns) | Delayed $\tau_2$ (ns) | a (%) | PL Count Ratio (Prompt : Delayed) $(1-a)\tau_1 : a\tau_2$ |
|---|---|---|---|---|
| **2-Cl** | 0.2 | 7.7 | 8 | 24 : 76 |
| **2-Me** | 0.3 | 86 | 10 | 3 : 97 |
| **2,6-Cl$_2$** | 0.3 | 92 | 11 | 3 : 97 |
| **2,6-Cl,Me** | 0.1 | 105 | 18 | 0.4 : 99.6 |
| **2,6-Me$_2$** | 0.1 | 95 | 22 | 0.4 : 99.6 |
| **Se-2,6-Cl$_2$** | 0.9 | 40 | 55 | 2 : 98 |
| **Se-2,6-Me$_2$** | 1.7 | 37 | 40 | 7 : 93 |

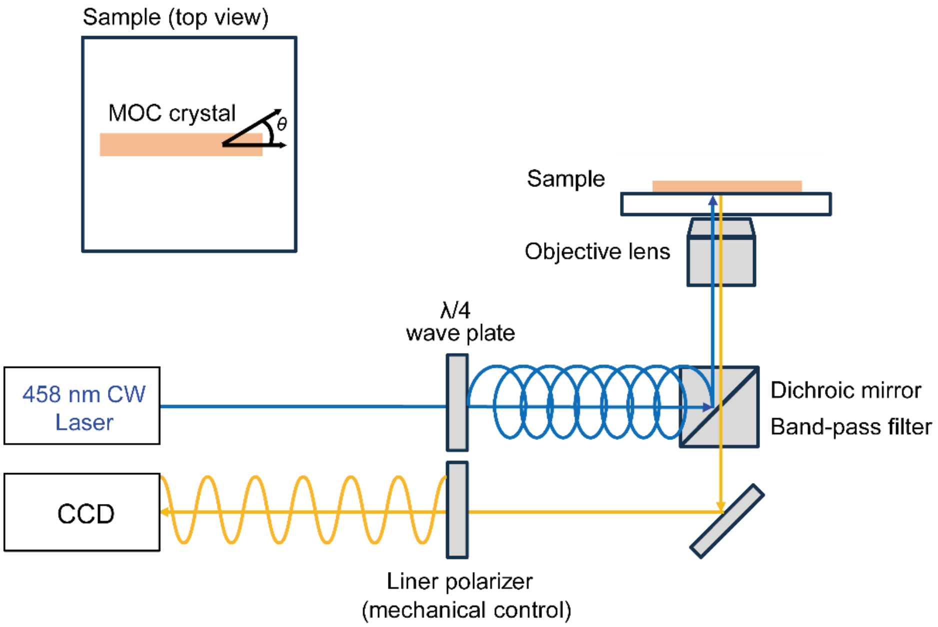


**Figure S13.** Schematic illustration of the experimental setup used for the polarized luminescence characterization of a single crystal.

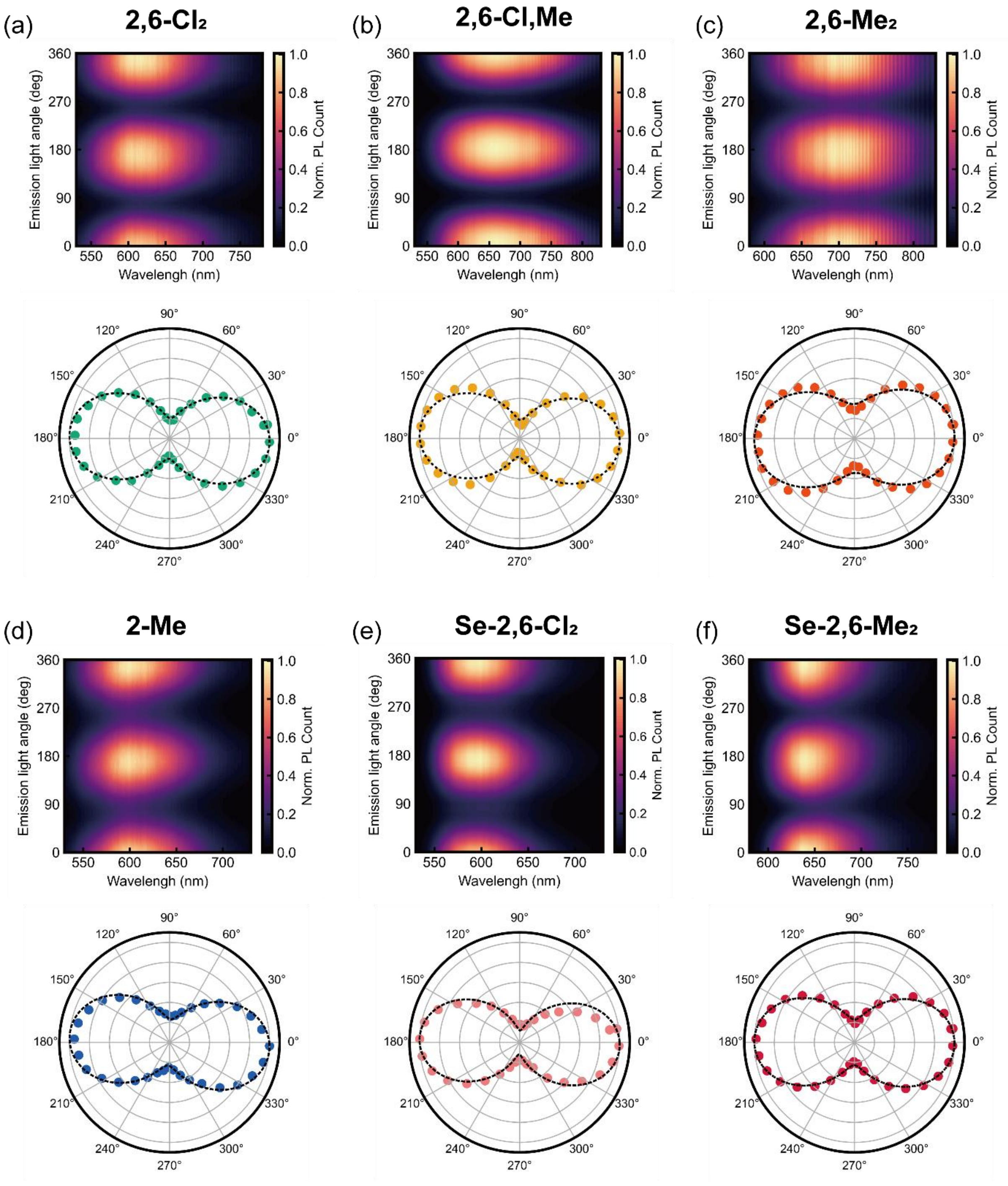


**Figure S14.** 2D polarization plots of PL intensity as a function of wavelength and polar plots at the PL peak position for (a) **2,6-Cl$_2$**, (b) **2,6-Cl,Me**, (c) **2,6-Me$_2$**, (d) **2-Me**, (e) **Se-2,6-Cl$_2$**, and (f) **Se-2,6-Me$_2$**.

**Table S9.** Carrier diffusion length of various materials[25–30]

| Group | Material | Diffusion length [nm] | Reference |
|---|---|---|---|
| Organic molecule (single crystal) | PTCDA | 25 | 24 |
| Organic molecule (single crystal) | BPTTE | 114.2 | 25 |
| Organic molecule (polycrystal) | Anthracene | 491 | 26 |
| Organic molecule (single crystal) | Rubrene | 3400 | 27 |
| Inorganic (0D, Quantum dots) | CdSe/CdS (core/shell) | 19-24 | 28 |
| Inorganic (1D) | SWCNT | 80-120 | 29 |

**Table S10.** Calculated effective masses of carriers and average diffusion coefficients and lengths.

| MOCs | $m_h$* ($m_0$) | $m_e$* ($m_0$) | Diffusion coefficient ($10^{-4}$ $cm^2/s$) | Diffusion length (nm) |
|---|---|---|---|---|
| **2-Me** | 0.52 | 2.00 | 5.08 ± 2.28 | 69.2 ± 18.5 |
| **2,6-Me₂** | 1.02 | 5.12 | 2.46 ± 1.25 | 52.4 ± 12.3 |
| **Se-2,6-Me₂** | 0.23 | 0.36 | 35.8 ± 26.1 | 128.1 ± 41.4 |

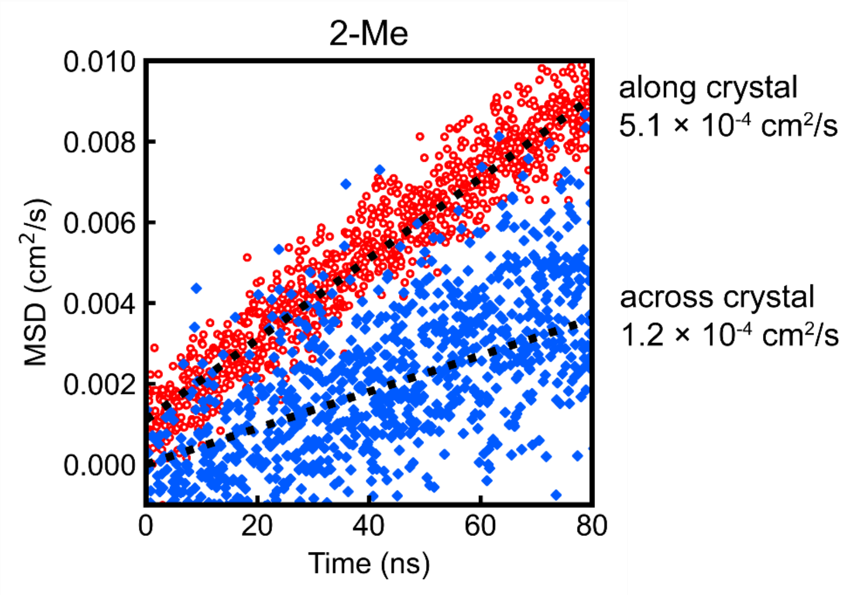


**Figure S15.** Time evolution of the mean squared displacement (MSD) of **2-Me** along the crystal orientation (red) and across the crystal orientation (blue).

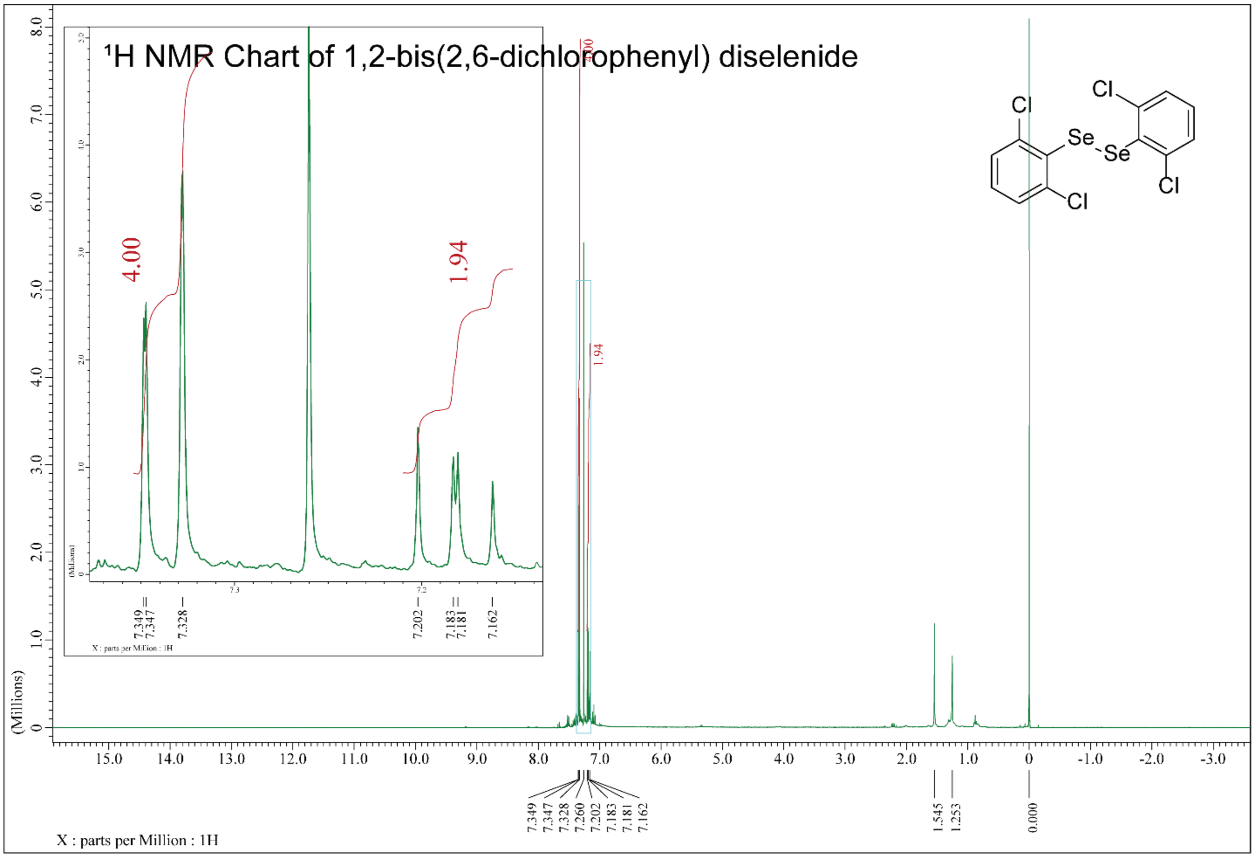

¹H NMR Chart of 1,2-bis(2,6-dichlorophenyl) diselenide
4.00
1.94
7.349
7.347
7.328
7.260
7.202
7.183
7.181
7.162
1.545
1.253
0.000
X : parts per Million : 1H
(Millions)


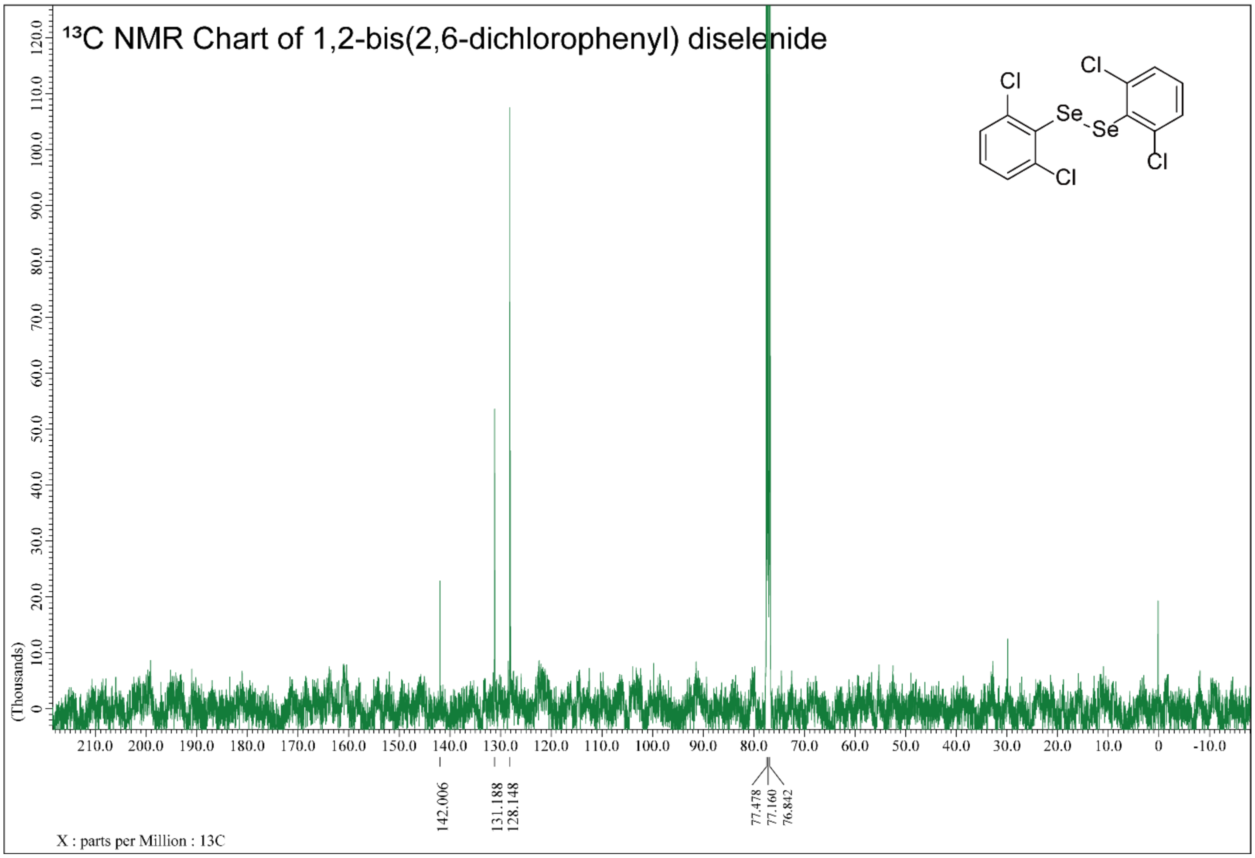

¹³C NMR Chart of 1,2-bis(2,6-dichlorophenyl) diselenide
142.006
131.188
128.148
77.478
77.160
76.842
X : parts per Million : 13C
(Thousands)

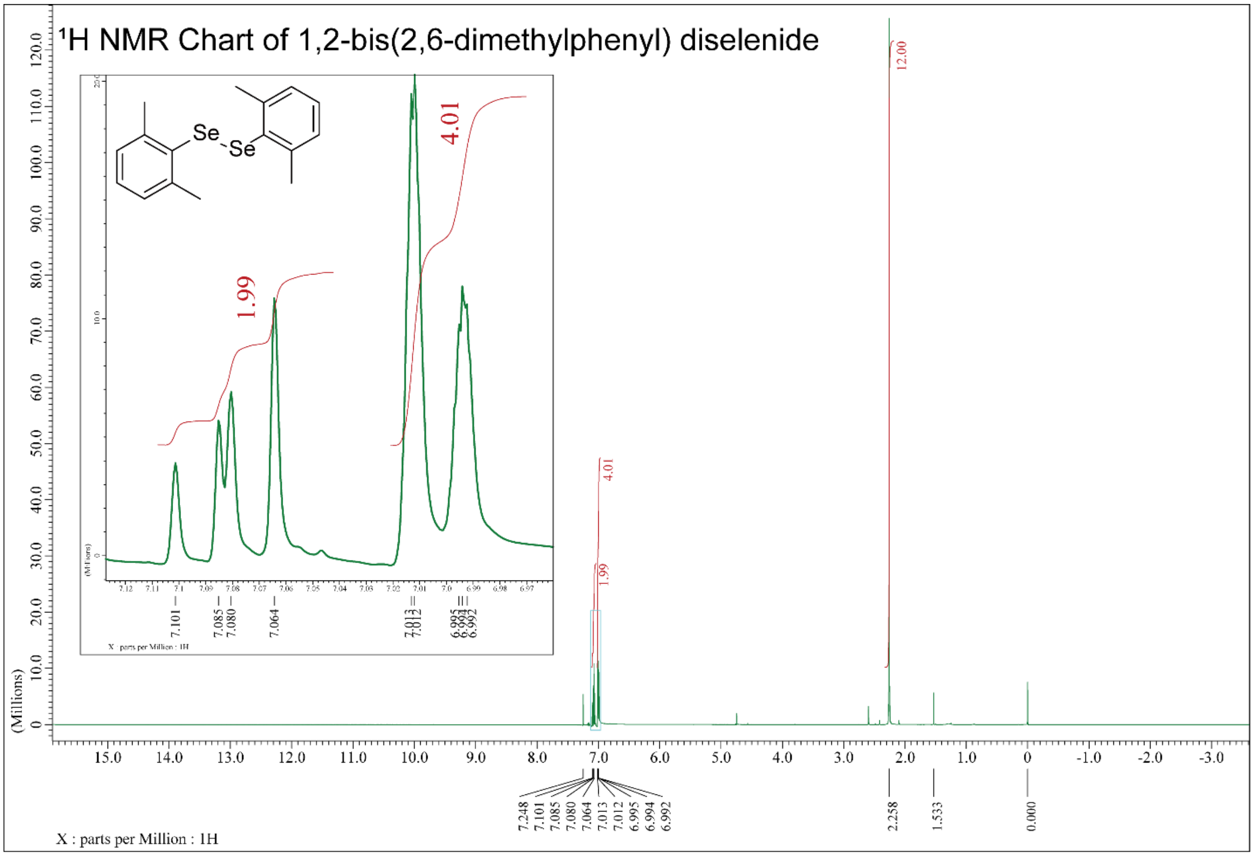

¹H NMR Chart of 1,2-bis(2,6-dimethylphenyl) diselenide
Se
Se
1.99
4.01
12.00
7.101
7.085
7.080
7.064
7.013
6.995
6.992
7.248
7.012
6.994
2.258
1.533
0.000
X : parts per Million : 1H
(Millions)


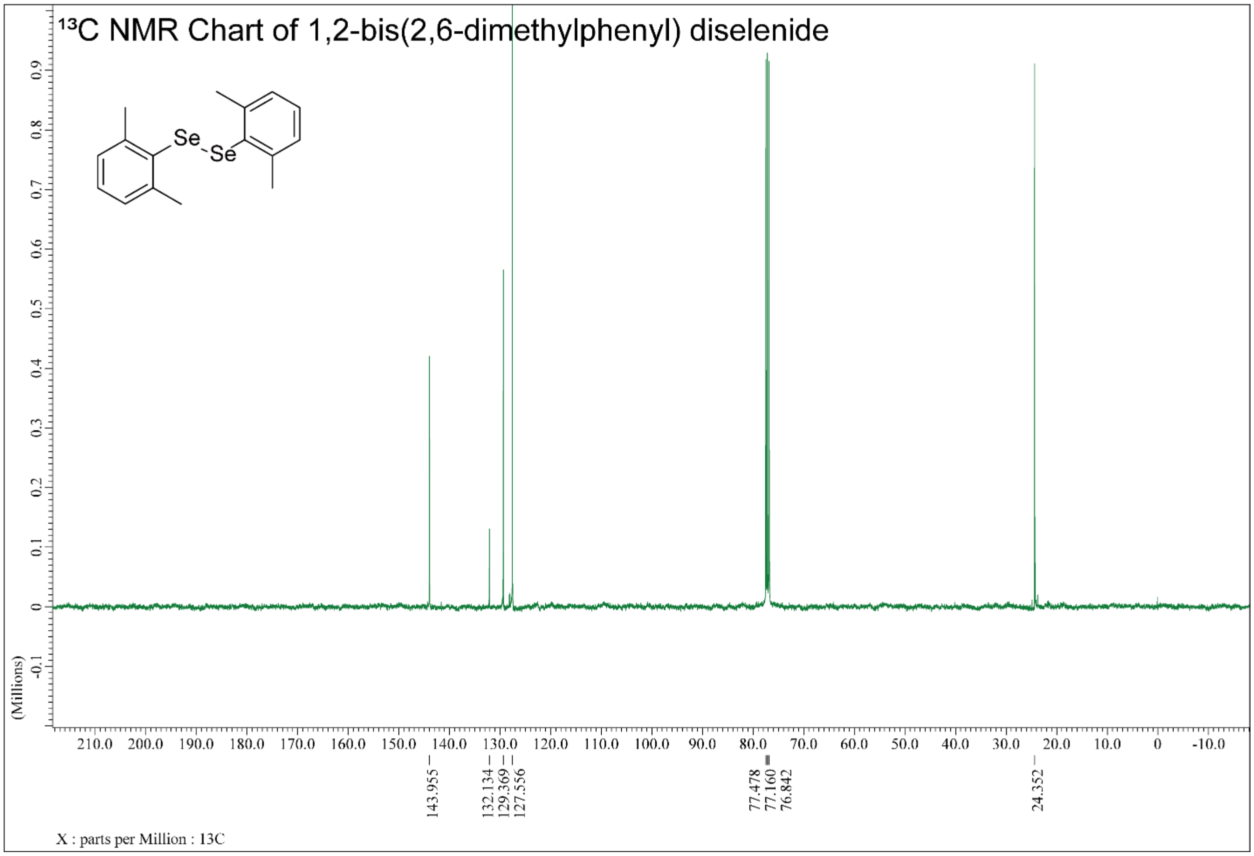

¹³C NMR Chart of 1,2-bis(2,6-dimethylphenyl) diselenide
Se
Se
143.955
132.134
129.369
127.556
77.478
77.160
76.842
24.352
X : parts per Million : 13C
(Millions)

## 4. References


(1) Kotei, P. A.; Paley, D. W.; Oklejas, V.; Mittan-Moreau, D. W.; Schriber, E. A.; Aleksich, M.; Willson, M. C.; Inoue, I.; Owada, S.; Tono, K.; Sugahara, M.; Inaba-Inoue, S.; Aquila, A.; Poitevin, F.; Blaschke, J. P.; Lisova, S.; Hunter, M. S.; Sierra, R. G.; Gascón, J. A.; Sauter, N. K.; Brewster, A. S.; Hohman, J. N. Engineering Supramolecular Hybrid Architectures with Directional Organofluorine Bonds. *Small Science* **2024**, *4* (1), 2300110. https://doi.org/10.1002/smsc.202300110.

(2) Abdallah, A.; Vaidya, S.; Hawila, S.; Ornis, S.-L.; Nebois, G.; Barnet, A.; Guillou, N.; Fateeva, A.; Mesbah, A.; Ledoux, G.; Bérut, A.; Vanel, L.; Demessence, A. Luminescent and Sustainable D10 Coinage Metal Thiolate Coordination Polymers for High-Temperature Optical Sensing. *iScience* **2023**, *26* (2). https://doi.org/10.1016/j.isci.2023.106016.

(3) Veselska, O.; Guillou, N.; Diaz-Lopez, M.; Bordet, P.; Ledoux, G.; Lebègue, S.; Mesbah, A.; Fateeva, A.; Demessence, A. Sustainable and Efficient Low-Energy Light Emitters: A Series of One-Dimensional D10 Coinage Metal–Organic Chalcogenolates, [M(o-SPhCO2H)]n. *ChemPhotoChem* **2022**, *6* (5), e202200030. https://doi.org/10.1002/cptc.202200030.

(4) Aleksich, M.; Cho, Y.; Paley, D. W.; Willson, M. C.; Nyiera, H. N.; Kotei, P. A.; Oklejas, V.; Mittan-Moreau, D. W.; Schriber, E. A.; Christensen, K.; Inoue, I.; Owada, S.; Tono, K.; Sugahara, M.; Inaba-Inoue, S.; Vakili, M.; Milne, C. J.; DallAntonia, F.; Khakhulin, D.; Ardana-Lamas, F.; Lima, F.; Valerio, J.; Han, H.; Gallo, T.; Yousef, H.; Turkot, O.; Macias, I. J. B.; Kluyver, T.; Schmidt, P.; Gelisio, L.; Round, A. R.; Jiang, Y.; Vinci, D.; Uemura, Y.; Kloos, M.; Mancuso, A. P.; Warren, M.; Sauter, N. K.; Zhao, J.; Smidt, T.; Kulik, H. J.; Sharifzadeh, S.; Brewster, A. S.; Hohman, J. N. Ligand-Mediated Quantum Yield Enhancement in 1-D Silver Organothiolate Metal–Organic Chalcogenolates. *Advanced Functional Materials* *n/a* (n/a), 2414914. https://doi.org/10.1002/adfm.202414914.

(5) Veselska, O.; Abdallah, A.; Giraudon, A.; Andrade, C.; Hawila, S.; Guillou, N.; Jeanneau, E.; Monge, M.; Mesbah, A.; Fateeva, A.; Pailhès, S.; Ledoux, G.; Perret, F.; Demessence, A. Effect of the 1D / 2D Dimensionality in Copper and Silver Thiolate Coordination Polymers on Their Photophysical Properties. *Chem. Commun.* **2024**. https://doi.org/10.1039/D4CC06171C.

(6) Veselska, O.; Dessal, C.; Melizi, S.; Guillou, N.; Podbevšek, D.; Ledoux, G.; Elkaim, E.; Fateeva, A.; Demessence, A. New Lamellar Silver Thiolate Coordination Polymers with Tunable Photoluminescence Energies by Metal Substitution. *Inorg. Chem.* **2019**, *58* (1), 99–105. https://doi.org/10.1021/acs.inorgchem.8b01257.

(7) Sakurada, T.; Cho, Y.; Paritmongkol, W.; Lee, W. S.; Wan, R.; Su, A.; Shcherbakov-Wu, W.; Müller, P.; Kulik, H. J.; Tisdale, W. A. 1D Hybrid Semiconductor Silver 2,6-Difluorophenylselenolate. *J. Am. Chem. Soc.* **2023**, *145* (9), 5183–5190. https://doi.org/10.1021/jacs.2c11896.

(8) Paritmongkol, W.; Sakurada, T.; Lee, W. S.; Wan, R.; Müller, P.; Tisdale, W. A. Size and Quality Enhancement of 2D Semiconducting Metal–Organic Chalcogenolates by Amine Addition. *J. Am. Chem. Soc.* **2021**, *143* (48), 20256–20263. https://doi.org/10.1021/jacs.1c09106.

(9) Kubelka, P.; Munk, F. An Article on Optics of Paint Layers. *Z. Technol. Phys.* **1931**, *12*, 593.

(10) Sheldrick, G. M. SHELXT – Integrated Space-Group and Crystal-Structure Determination. *Acta Cryst A* **2015**, *71* (1), 3–8. https://doi.org/10.1107/S2053273314026370.

(11) Dolomanov, O. V.; Bourhis, L. J.; Gildea, R. J.; Howard, J. a. K.; Puschmann, H. OLEX2: A Complete Structure Solution, Refinement and Analysis Program. *J Appl Cryst* **2009**, *42* (2), 339–341. https://doi.org/10.1107/S0021889808042726.

(12) Sheldrick, G. M. Crystal Structure Refinement with SHELXL. *Acta Cryst C* **2015**, *71* (1), 3–8. https://doi.org/10.1107/S2053229614024218.

(13) Khamlue, R. *et al.* Chelation-Driven Dissolution and Single-Crystal Growth of Hybrid Metal Organochalcogenide Semiconductors by Poly-Dentate Amines. **2025**, *submitted*.

(14) Kresse, G.; Furthmüller, J. Efficient Iterative Schemes for Ab Initio Total-Energy Calculations Using a Plane-Wave Basis Set. *Phys. Rev. B* **1996**, *54* (16), 11169–11186. https://doi.org/10.1103/PhysRevB.54.11169.

(15) Kresse, G.; Furthmüller, J. Efficiency of Ab-Initio Total Energy Calculations for Metals and Semiconductors Using a Plane-Wave Basis Set. *Computational Materials Science* **1996**, *6* (1), 15–50. https://doi.org/10.1016/0927-0256(96)00008-0.
(16) Kresse, G.; Hafner, J. Ab Initio Molecular Dynamics for Liquid Metals. *Phys. Rev. B* **1993**, *47* (1), 558–561. https://doi.org/10.1103/PhysRevB.47.558.
(17) Kresse, G.; Joubert, D. From Ultrasoft Pseudopotentials to the Projector Augmented-Wave Method. *Phys. Rev. B* **1999**, *59* (3), 1758–1775. https://doi.org/10.1103/PhysRevB.59.1758.
(18) Perdew, J. P.; Burke, K.; Ernzerhof, M. Generalized Gradient Approximation Made Simple. *Phys. Rev. Lett.* **1996**, *77* (18), 3865–3868. https://doi.org/10.1103/PhysRevLett.77.3865.
(19) Becke, A. D. A New Mixing of Hartree–Fock and Local Density-Functional Theories. *The Journal of Chemical Physics* **1993**, *98* (2), 1372–1377. https://doi.org/10.1063/1.464304.
(20) Grimme, S.; Antony, J.; Ehrlich, S.; Krieg, H. A Consistent and Accurate Ab Initio Parametrization of Density Functional Dispersion Correction (DFT-D) for the 94 Elements H-Pu. *The Journal of Chemical Physics* **2010**, *132* (15), 154104. https://doi.org/10.1063/1.3382344.
(21) Grimme, S.; Ehrlich, S.; Goerigk, L. Effect of the Damping Function in Dispersion Corrected Density Functional Theory. *Journal of Computational Chemistry* **2011**, *32* (7), 1456–1465. https://doi.org/10.1002/jcc.21759.
(22) Kane, E. O. Band Structure of Indium Antimonide. *Journal of Physics and Chemistry of Solids* **1957**, *1* (4), 249–261. https://doi.org/10.1016/0022-3697(57)90013-6.
(23) Whalley, L. D.; Frost, J. M.; Morgan, B. J.; Walsh, A. Impact of Nonparabolic Electronic Band Structure on the Optical and Transport Properties of Photovoltaic Materials. *Phys. Rev. B* **2019**, *99* (8), 085207. https://doi.org/10.1103/PhysRevB.99.085207.
(24) Whalley, L. D. Effmass: An Effective Mass Package. *Journal of Open Source Software* **2018**, *3* (28), 797. https://doi.org/10.21105/joss.00797.
(25) Lunt, R. R.; Benziger, J. B.; Forrest, S. R. Relationship between Crystalline Order and Exciton Diffusion Length in Molecular Organic Semiconductors. *Advanced Materials* **2010**, *22* (11), 1233–1236. https://doi.org/10.1002/adma.200902827.
(26) Zhang, Y.; Yuan, Y.; Wang, P.; Gao, H.; Qin, Z.; Liu, D.; Zhang, X.; Gao, C.; Fang, H.-H.; Hu, W.; Dong, H. Long Exciton Diffusion Length in High Mobility Emissive Organic Semiconductor. *J. Phys. Chem. C* **2024**, *128* (8), 3431–3437. https://doi.org/10.1021/acs.jpcc.4c00234.
(27) Narushima, K.; Hirata, S.; Vacha, M. Nanoscale Triplet Exciton Diffusion via Imaging of Up-Conversion Emission from Single Hybrid Nanoparticles in Molecular Crystals. *Nanoscale* **2017**, *9* (30), 10653–10661. https://doi.org/10.1039/C7NR01569K.
(28) Narushima, K.; Kiyota, Y.; Mori, T.; Hirata, S.; Vacha, M. Suppressed Triplet Exciton Diffusion Due to Small Orbital Overlap as a Key Design Factor for Ultralong-Lived Room-Temperature Phosphorescence in Molecular Crystals. *Advanced Materials* **2019**, *31* (10), 1807268. https://doi.org/10.1002/adma.201807268.
(29) Lee, E. M. Y.; Tisdale, W. A. Determination of Exciton Diffusion Length by Transient Photoluminescence Quenching and Its Application to Quantum Dot Films. *J. Phys. Chem. C* **2015**, *119* (17), 9005–9015. https://doi.org/10.1021/jp512634c.
(30) Cognet, L.; Tsyboulski, D. A.; Rocha, J.-D. R.; Doyle, C. D.; Tour, J. M.; Weisman, R. B. Stepwise Quenching of Exciton Fluorescence in Carbon Nanotubes by Single-Molecule Reactions. *Science* **2007**, *316* (5830), 1465–1468. https://doi.org/10.1126/science.1141316.
